# Development of a genus-specific next generation sequencing approach for sensitive and quantitative determination of the *Legionella* microbiome in freshwater systems

**DOI:** 10.1186/s12866-017-0987-5

**Published:** 2017-03-31

**Authors:** Rui P. A. Pereira, Jörg Peplies, Ingrid Brettar, Manfred G. Höfle

**Affiliations:** 1 Department of Vaccinology and Applied Microbiology, RG Microbial Diagnostics, Helmholtz Centre for Infection Research (HZI), Inhoffenstr. 7, 38124 Braunschweig, Germany; 2 Ribocon GmbH, Fahrenheitstraße 1, 28359 Bremen, Germany; 3 Present address: School of Life Sciences, University of Warwick, Coventry, CV4 7AL UK

**Keywords:** Molecular diagnostics, Drinking water, Cooling tower, Pathogen profiling, Patho-microbiome

## Abstract

**Background:**

Next Generation Sequencing (NGS) has revolutionized the analysis of natural and man-made microbial communities by using universal primers for bacteria in a PCR based approach targeting the 16S rRNA gene. In our study we narrowed primer specificity to a single, monophyletic genus because for many questions in microbiology only a specific part of the whole microbiome is of interest. We have chosen the genus *Legionella,* comprising more than 20 pathogenic species, due to its high relevance for water-based respiratory infections.

**Methods:**

A new NGS-based approach was designed by sequencing 16S rRNA gene amplicons specific for the genus *Legionella* using the Illumina MiSeq technology. This approach was validated and applied to a set of representative freshwater samples.

**Results:**

Our results revealed that the generated libraries presented a low average raw error rate per base (<0.5%); and substantiated the use of high-fidelity enzymes, such as KAPA HiFi, for increased sequence accuracy and quality. The approach also showed high *in situ* specificity (>95%) and very good repeatability. Only in samples in which the gammabacterial clade SAR86 was present more than 1% non-Legionella sequences were observed. Next-generation sequencing read counts did not reveal considerable amplification/sequencing biases and showed a sensitive as well as precise quantification of *L. pneumophila* along a dilution range using a spiked-in, certified genome standard. The genome standard and a mock community consisting of six different *Legionella* species demonstrated that the developed NGS approach was quantitative and specific at the level of individual species, including *L. pneumophila*. The sensitivity of our genus-specific approach was at least one order of magnitude higher compared to the universal NGS approach. Comparison of quantification by real-time PCR showed consistency with the NGS data. Overall, our NGS approach can determine the quantitative abundances of *Legionella* species, i. e. the complete *Legionella* microbiome, without the need for species-specific primers.

**Conclusions:**

The developed NGS approach provides a new molecular surveillance tool to monitor all *Legionella* species in qualitative and quantitative terms if a spiked-in genome standard is used to calibrate the method. Overall, the genus-specific NGS approach opens up a new avenue to massive parallel diagnostics in a quantitative, specific and sensitive way.

**Electronic supplementary material:**

The online version of this article (doi:10.1186/s12866-017-0987-5) contains supplementary material, which is available to authorized users.

## Background

For many aspects of microbiology only part of the microbiome is of specific interest. For instance, in medical and environmental diagnostics the prime interest often lays in pathogenic or potentially pathogenic microorganisms. We will call this part of the microbiome “patho-microbiome” and it can consist of viruses, bacteria and lower eukaryotes. Despite the importance of assessing the total patho-microbiome, the methodologies to address all major pathogenic microorganisms are very complex [1]. To make such an enterprise more amenable to a straight forward methodological approach, we have chosen the genus *Legionella* as a subject, which is not only relevant for medical and environmental microbiologists but also taxonomically well defined.

Within the genus *Legionella*, several described species can cause clinical disease in humans, such as *L. bozemanii*, *L. longbeachae*, *L. micdadei* and *L. pneumophila* [2, 3]. Nonetheless, the latest, i.e. *L. pneumophila*, is the most common causative agent of legionellosis (about 90% of the cases worldwide) or Legionnaires’ disease, the majority of them community-acquired [4]. The infections caused by almost all non-*L. pneumophila* species have been associated with freshwater systems in hospital settings [3]*.* Natural aquatic environments are the major reservoir for *Legionella* species, with aerosol-generating man-made water systems such as cooling towers, drinking water supply systems (DWSS) and recreational waters being the main sources of human exposure to *Legionella* species [5]. Exceptionally, *L. longbeachae* infections are commonly linked with the presence of the bacteria in potting soils, instead of freshwater environments, with a high frequency of reported cases in Australia [6].

In freshwater systems, monitoring of pathogenic bacteria, hazard prediction and risk assessment are of high relevance to human health. To successfully achieve this, rapid and accurate detection of clinically relevant pathogens and information on bacterial diversity in contaminated systems are needed in order to improve prevention and achieve a better pathogen control. Currently, there are several methods for detection and/or quantification of *Legionella* species in freshwater systems including: culture, fluorescent *in situ* hybridization (FISH), flow cytometry, endpoint PCR and qPCR assays [7]. Though EU governmental agencies request cultivation as the official reference standard method, several limitations have been reported, such as the need of complex culture media and relatively long incubation times for *Legionella* growth; the overgrowth of competing and unwanted organisms; and the ability of *Legionella* species to undergo a viable but non-culturable (VBNC) state [8, 9]. To overcome these limitations, molecular assays have been developed to provide rapid, highly specific and sensitive detection and quantification of *Legionella* species [10, 11].

Advances in molecular anaylsis in the last decade have provided new opportunities to approach and improve diagnosis of pathogens. Next-Generation Sequencing (NGS) will be more frequently applied for the assessment of microbial water quality with the expectation to understand which part of the DWSS and which pathogens are critical to human health [12]. Especially, sequencing of PCR amplicons of 16S rRNA genes has been very successfully used to understand community dynamics of the drinking water microbiome [13]. Thus, high-throughput sequencing of PCR amplicons has the potential to provide valuable information regarding not only the environmental distribution and diversity of *Legionella* species but also the temporal and spatial behaviour of the whole *Legionella* microbiome. To this end, we pursued a new NGS approach for the assessment of the *Legionella* microbiome based on the genus-specific amplification of 16S rRNA genes using the Illumina MiSeq technology. Such an approach should be validated with environmental DNA obtained from reference water samples of several freshwater environments, such as cold and hot drinking water and water from a cooling tower. Genome standards for *L. pneumophila* and other *Legionella* species should be used to calibrate the molecular assay and determine its accuracy and precision. Along these lines, the aim of our study was to develop and validate a *Legionella* genus-specific Illumina-based NGS approach that allows accurate identification and precise quantification of the most important *Legionella* species, i. e. the *Legionella* microbiome. Validation of the developed approach was done *in silico*, in vitro and *in situ*, with emphasis on the latter by using a set of representative freshwater samples including a comparison with real-time PCR measurements in terms of the accuracy of quantification of *Legionella* genus-specific abundances by the NGS-based approach.

## Methods

### Freshwater systems and sampling

Cold drinking water was sampled in March and April 2009. It was taken from the tap of laboratory D0.04 on the campus of the Helmholtz Centre for Infection Research (HZI), Braunschweig, Germany, with five minute flushing to prevent stagnant water to be sampled. This drinking water originates from two surface water reservoirs situated in the Harz Mountains, 40 km south of Braunschweig. More details on the respective drinking water supply system are given elsewhere [13]. Hot drinking water was sampled, in March and April 2009, from a shower next to laboratory D0.04 also with several minutes flushing. The protocol is described in more detail in a preceding publication [14]. Cooling tower water was monthly sampled between January 2013 and December 2014 from a cooling tower on the campus of the HZI. The studied cooling tower is used for discharge of waste heat generated by air conditioning and other heat producing processes at the site. It is an open circuit cooling tower with antifouling coating (Gohl GmbH, Singen, Germany). Regular drinking water is used as make up water, whereas blow down is an automated process based on the conductivity of the water. Disinfection of the system was achieved by continuous silver plus hydrogen peroxide treatment.

Bulk water microorganisms were sampled by filtration according to Eichler et al. [13]. Briefly, 5 l of drinking water and 3 l of cooling tower water were filtered through a filter sandwich consisting of a 0.2 μm pore size polycarbonate filter (90 mm diameter; Nucleopore; Whatman, Maidstone, UK) with a precombusted glass fiber filter on top (90 mm diameter; GF/F; Whatman, Maidstone, UK). Biomass harvested on filter sandwiches was stored at−70 °C for the molecular analyses.

### DNA extraction of water samples

DNA was extracted from the filter sandwiches according to a modified DNeasy (Qiagen, Hilden, Germany) protocol by Henne et al. [14]. This DNA extraction protocol consists of cutting sandwich filters into pieces and an incubation step with lysis buffer containing 10 mg/ml lysozyme (Sigma) for 60 min at 37 °C. Next, samples were digested with proteinase K digestion according to the manufacturer instructions and heated to 70 °C in a water bath for 20 min. After filtration through a polyamide mesh with 250 μm mesh size, absolute ethanol was added to the filtrate (ratio filtrate/ethanol 2:1) and the mixture was applied to the adequate spin-column of the Qiagen kit. From this point forward, the washing and elution steps were performed according to the manufacturer instructions. DNA extracts were stored at−20 °C until further molecular analyses.

### Illumina MiSeq library design and preparation

NGS libraries construction and preparation were adapted from previously described protocols [15–18]. A dual-indexing strategy was chosen and primer sequences are described in the supplementary material (Additional file 1: Table S1). More specifically, the amplification of a 421 bp region of the 16S rRNA gene of *Legionella* including the variable regions V3 and V4 was performed using primers Lgsp17F 5’-GGCCTACCAAGGCGACGATCG-3’ (*Escherichia coli* 16S rRNA gene position 264–284) and Lgsp28R 5’-CACCGGAAATTCCACTACCCTCTC-3’ (*E. coli* 16S rRNA gene starting position 661–684), previously developed by Kahlisch et al. [19]. The *Legionella* genus-specific primer pair Lgsp17F/Lgsp28R was then combined with the indexing primers for multiplexing and sequencing on the Illumina MiSeq platform. All primers were synthesized and HPSF purified by Eurofins MWG Operon, Ebersberg, Germany. The 16S rRNA gene amplicons (details of PCR conditions see next paragraph) were size-selected by gel electrophoresis on a 2% agarose gel pre-stained with GelRed Nucleic Acid Gel Stain (Biotium, Hayward, CA, USA). The bands with the expected target length were extracted over an UV light box and then recovered using the QIAquick Gel Extraction Kit (Qiagen, Hilden, Germany). QIAquick Gel Extraction Kit manufacturer’s specifications were slightly modified. Briefly, 1.5 ml of Buffer QG was added to the gel band followed by incubation at room temperature. Next, 250 μl of isopropanol (AppliChem, Darmstadt, Germany) were added, the mixture vortexed and placed in a QIAquick spin column and centrifuged for 1 min at 13,000 rpm. The remaining steps were performed according to the manufacturer instructions to obtain clean library amplicons, concentrated in 30 μl of EB buffer. DNA concentration of the extracted amplicons was assessed on a VICTOR X3 2030 Multilabel Plate Reader (Perkin Elmer, Germany) using a Quant-iT Picogreen dsDNA assay kit (Life Technologies, Oregon, USA) to ensure, when required, equimolar amounts of DNA with unique indices were pooled. Subsequently, pooled samples were purified by the MinElute PCR Purification Kit according to manufacturer protocol (Qiagen, Hilden, Germany). Molarity was quantified and library fragment size confirmed with Agilent Bioanalyzer. The resulting libraries were sequenced, in the Genome Analysis Department of the HZI, using Illumina MiSeq platform, generating paired-end 250 bp sequences.

### Amplification and Sequencing of *Legionella*-specific 16S rRNA fragment using KAPA HiFi DNA polymerase and HotStarTaq DNA polymerase

We compared the proof reading DNA polymerase KAPA HiFI with the most widely used HotStarTaq DNA polymerase to understand the effect of the DNA polymerase on the sequencing error rate. The *Legionella*-specific PCR reaction, using KAPA HiFi DNA polymerase, had the following components (50 μl total volume): KAPA HiFi HotStart ReadyMix 1X (Peqlab, Erlangen, Germany), 0.3 μM of each primer and 5 ng of the extracted environmental DNA. The PCR was carried out on a Biorad Thermo cycler 96-well iCycler under the following conditions: an initial denaturation step of 95° for 5 min, followed by 30 cycles of 98 °C for 20 s, 68 °C for 20 s, 72 °C for 20 s, with a final extension at 72 °C for 5 min. A second PCR (Multiplexing) had the following components (50 μl total volume): KAPA HiFi HotStart ReadyMix 1X (Peqlab, Erlangen, Germany), 0.3 μM of each primer and 2 μl of the previous PCR amplicons. This PCR was carried out on a Biorad Thermo cycler 96-well iCycler under the following conditions: an initial denaturation step of 95 °C for 5 min, followed by 10 cycles of 98 °C for 20 s, 59 °C for 20 s, 72 °C for 20 s, with a final extension at 72 °C for 7 min.

For HotStarTaq DNA polymerase-based amplification, the PCR mix comprised 0.1 mM concentration of each dNTP (Bioline, Luckenwalde, Germany), 0.4 mM MgCl_2_, 1X PCR reaction buffer, 0.03 U of HotStarTaq DNA polymerase (Qiagen, Hilden, Germany), 0.4 μM of each primer and 5 ng of the extracted environmental DNA in a total volume of 50 μl. The PCR included an initial denaturation step of 95 °C for 15 min; 30 cycles of 1 min at 95 °C, 30 s at 66.5 °C and 30 s at 72 °C followed by a final extension at 72 °C for 10 min. 2 μL of the target-specific PCR product from the previous PCR reaction were used as template for a second PCR reaction (Multiplexing PCR). Additionally, the mix contained 0.1 mM concentration of each dNTP (Bioline, Luckenwalde, Germany), 0.75 mM MgCl_2_, 1X PCR reaction buffer, 0.03 U of HotStarTaq DNA polymerase (Qiagen, Hilden, Germany), 0.4 μM of each primer. The PCR conditions included a cycle at 95 °C for 15 min, 10 cycles of 95 °C for 45 s, 57 °C for 30 s and 72 °C for 30 s, ending with an extension at 72 °C for 10 min.

### Pan-bacterial amplification and sequencing of 16S rRNA gene

We applied a commonly used PCR protocol with universal primers [20] for a comparison of the *Legionella* genus-specific sequences and their read abundances with the ones obtained from all *Bacteria*. In this universal PCR the variable regions V4 and V5 of the 16S rRNA gene of *Bacteria* were amplified using the modified universal primers 519 F (5’-CAGCAGCCGCGGTAATAC-3’) and 907R (5’-CCGTCAATTCCTTTGAGTTT-3’) [20]. All reactions were carried out with 50 μl reaction mixtures. The target-specific PCR mix comprised 0.1 mM concentration of each dNTP (Bioline, Luckenwalde, Germany), 0.4 mM MgCl_2_, 1X PCR reaction buffer, 0.03 U of HotStarTaq Polymerase (Qiagen, Hilden, Germany), 0.4 μM of each primer and 1 ng of environmental DNA. Cycling was performed on a Biorad Thermo cycler 96-well iCycler with an initial denaturation step at 95 °C for 15 min; 30 cycles of 1 min at 95 °C, 40 s at 55 °C, 40 s at 72 °C followed by a final extension at 72 °C for 10 min. The multiplexing PCR was performed as described above for the HotStarTaq DNA polymerase-based amplification and the primers were analogous to the ones provided in Additional file 1: Table S1, i.e. the primer pair Lgsp17F/Lgsp28R was replaced by primers 519 F/907R.

### Assessment of sensitivity and quantitative precision of NGS

For the assessment of sensitivity of NGS and its potential for absolute quantification of *Legionella* species a certified standard of genomic DNA was used. For these spike-in experiments ten-fold dilutions of a molecular standard based on genomic DNA of *L. pneumophila* ATCC 33152^T^ (GPS-Genetic PCR Solutions, Alicante, Spain) ranging from 10^5^ to 10^1^ genome copies were added to triplicate 5 ng environmental DNA aliquots of a drinking water sample. The sample without spiked *L. pneumophila* was also analysed. In addition, the same range of *L. pneumophila* was spiked into nuclease free water to verify the existence of amplification inhibition and interference of bacterial community in the quantification of *L. pneumophila* in the drinking water sample. All samples were amplified by the *Legionella* genus-specific assay and after pooling of equal volumes of NGS libraries, sequenced with Illumina MiSeq platform.

For the determination of specificity and precision of the relative abundance assessment, a mock community was constituted by adding equivalent concentrations of 16S rDNA of the following nine strains: *L. anisa* ATCC 35292^T^, *L. bozemanii* ATCC 33217^T^, *L. feelei* ATCC 35072^T^, *L. longbeachae* ATCC 33462^T^, *L. micdadei* ATCC 33218^T^, *L. pneumophila* ATCC 33152^T^, *Acinetobacter baumannii* DSM 30008, *Klebsiella pneumoniae* DSM 30104 and *Pseudomonas aeruginosa* DSM 50071^T^. Technical replicates (triplicates) of the defined synthetic community were amplified by the described *Legionella* genus-specific protocol and sequenced by the Illumina MiSeq platform.

### Determination of sequence accuracy and quality

The error rate of sequence reads was obtained for NGS assays with HotStarTaq and KAPA HiFi DNA polymerases by using internal controls consisting of nuclease-free water samples spiked in with genomic DNA of *L. pneumophila* ATCC 33152^T^ (GPS-Genetic PCR Solutions, Alicante, Spain). Technical triplicates were used. 5,000 paired-end merged reads of each triplicate were statistically sampled, for a total of 15,000 paired-end merged reads, and an alignment algorithm was applied against the *L. pneumophila* reference sequence, allowing detection of nucleotide substitutions, indels and unknown nucleotides, and subsequent calculation of the respective error rates.

### Data processing, clustering and taxonomic assignment

The pre-processing of Illumina MiSeq reads, i.e. merging of paired-end reads and barcode-based de-multiplexing, was done using Mothur software package (v.1.34.0) [21]. Reads were quality controlled and aligned with the bioinformatics pipeline of the SILVA project [22]. Each read was aligned using SINA (v1.2.10) [23]. Reads shorter than 200 aligned nucleotides and reads with more than 2% of ambiguities or 2% of homopolymers were excluded for downstream analysis. Reads with a low alignment quality (50 alignment identity, 40 alignment score reported by SINA), suggesting putative contaminations and artefacts, were also removed. Next, filtered reads were dereplicated, clustered and binned/classified. Dereplication (identification of identical reads ignoring overhangs) was done using cd-hit-est [24] using identity criterion level of 1.00. Clustering was also completed with cd-hit-est using identity criterion of 0.98. The representative sequence of each OTU was then classified using BLAST+ (version 2.2.28+) [25] against the non-redundant version of the SILVA SSU 115 NR dataset [26] with standard settings. Reads presenting a best blast hit values below the value 0.93 for the function (% sequence identity + % alignment coverage)/2 were not classified. For species-level classification, additional data processing steps were performed. A conservative approach with removal of singletons, doubletons, tripletons and quadrupletons, was pursued because application of chimera checking on low diversity genus-specific libraries is not very effective [27]. Filtered OTU reference sequences after trimming of primer sequence were blasted using BLASTN+ (version 2.2.28+) [25] against a database including curated and truncated (only V3-V4 region amplified) 16S rRNA sequences of *Legionella* species that were retrieved from the non-redundant version of the SILVA SSU 115 NR dataset. OTU reference sequences were assigned to the species level when sequence identity was higher or equal to 97%. This threshold was defined after amplification and sequencing of *L. pneumophila* ATCC 33152^T^ with HotStarTaq and KAPA HiFi DNA polymerases (Additional file 1: Figure S1). Our results revealed that at a 97% threshold more than 98% of the sequences generated were identified as *L. pneumophila*. For both acknowledged species and sequence clusters not assigned to a known species, the term phylotype will be used throughout the study.

### Quantification of *Bacteria* and *Legionella* microbiome by real-time PCR

For quantitative real-time PCR of the *Bacteria* the same 519 F/907R primers were used as for the NGS approach. For this real-time PCR the same calibration standard and DNA extracts were applied as above. The PCR mixture (20 μl total) contained 10 μl of LC480 SYBR Green Master Mix (2x, Roche Diagnostics, Mannheim, Germany), 1.6 μl pan-bacterial primers (final concentration: 0.8 μM each) and 2 to 4 ng of environmental DNA in 2 μl of PCR grade water. An initial denaturing step at 95 °C for 5 min was followed by 45 cycles of 95 °C for 30 s, 60 °C for 30 s and 72 °C for 30 s.

*Legionella* species abundance was quantified using 16S rRNA gene primers Lgsp17F/Lgsp28R as for the NGS library preparation. The qPCR reaction consisted of a total volume of 20 μl reaction mixture containing 10 μl of Master Mix as above, 2 μl of each primer (final concentration: 0.5 μM each, Eurofins MWG Operon, Ebersberg, Germany) and 1 to 5 ng of template DNA. Amplification cycling conditions consisted of an initial 5 min cycle at 95 °C followed by 45 cycles of 30 s at 95 °C, 60 °C and 72 °C each. All real-time PCR assays were run on a Light Cycler 480 (Roche Diagnostics, Mannheim, Germany) and potential contaminations and development of primer dimers were determined by melting curve analyses.

### Statistical analyses

The statistical comparison of two datasets was performed using parametric paired *t*-test, or Welch’s *t*-test in case of unequal variances. The null-hypothesis was rejected when *P*-value <0.05. The correlation between two variables was determined by Spearman rank of correlation (r_s_), or Pearson correlation (r) in case of linear relationship. T-tests and correlations were performed with Past 3 (version 3.09) [28]. Rarefaction curves to evaluate sequencing depth were generated using Analytic Rarefaction 1.3 [29].

Alpha-diversity metrics and Good’s coverage were calculated with software package Explicet [30]. Multivariate analyses were performed using PRIMER (Version 7.0.7) [31]. Bray-Curtis similarity [32] matrices were generated, by comparing the standardised, untransformed abundances of phylotypes, and represented by non-metric multidimensional scaling (MDS) plots. Sample cluster analysis was performed with SIMPROF [33], whereas potentially significant differences between groups of samples were determined using ANOSIM [33].

## Results

### Sequence accuracy and quality of generated NGS libraries

In order to minimise error rate, validation of our deep sequencing approach was performed with proofreading DNA polymerase KAPA HiFi and compared with the extensively used HotStarTaq DNA polymerase. With this purpose, we investigated the type, frequency and distribution of errors that occur during the amplification and sequencing steps. After NGS analysis of DNA from the *L. pneumophila* genome standard in nuclease-free water 6,315,000 bases of sequence information was compared. These sequences had an average error rate of 0.383% ± 0.28% that was significantly lower when compared to HotStarTaq enzyme (0.446% ± 0.283%) (*T*-test, *P* < 0.05). Erroneous sequencing was observed in all the 421 bases, with both enzymes, indicating no error-free base position in the sequences amplified. The minimum and maximum error frequencies per base with KAPA HiFi were lower than with HotStarTaq (0.033% and 1.971% against 0.067% and 2.159%) Additionally to a lower minimum, mean and maximum error frequency values per position, 65.1% of the sequences analysed were identified as error-free with KAPA HiFi, while, with HotStarTaq, this number was reduced to only 39.3% (Additional file 1: Figure S1).

When breaking down the error rate by error type, substitutions were the foremost, representing 84.1% of the total errors detected in the libraries generated by KAPA HiFi (Additional file 1: Figure S2a). The 16S rRNA sequences had an average substitution rate per base of 0.322% ± 0.277%, which represents a significant accuracy improvement to libraries amplified by HotStarTaq DNA polymerase (0.383% ± 0.275%) (*T*-test, *P* < 0.05). Unknown bases (Ns) were the next most frequent errors and had an average rate per base of 0.032% with KAPA HiFi and 0.035% with HotStarTaq. Deletions and insertions made a smaller contribution to the global error rate. Insertions with KAPA HiFi and HotStarTaq had an average rate per base of 0.011% and 0.008%, respectively. A comparison of the substitution error profiles between enzymes showed different patterns in relation to individual bases (Additional file 1: Figure S2b). With HotStarTaq, transitions and transversions were about equally represented. However, with KAPA HiFi, transversions were clearly more frequent accounting for 67.1% of all substitutions. This imbalance between transitions and transversions is mostly due to a significant reduction of 46.3% in the number of transitions with KAPA HiFi (*T*-test, *P* < 0.05).

**Fig. 1:**
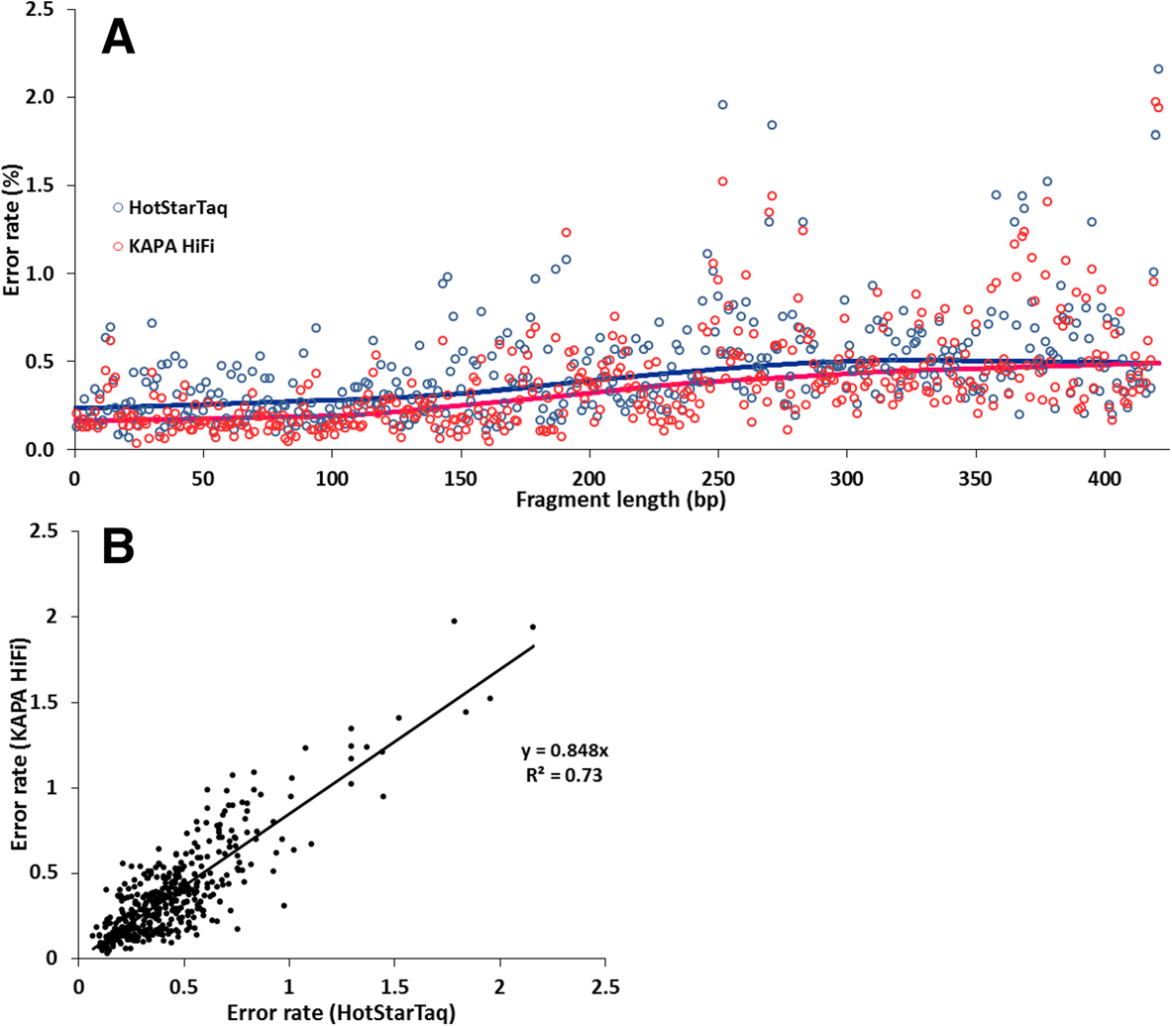
Sequencing error rate distribution. **a)** Error rate distribution along the full-length fragment (421 bp) after analysis of 15,000 reads of *L. pneumophila* amplified with HotStarTaq (*open blue circles*) and KAPA HiFi DNA polymerases (*red open circles*). LOESS curves illustrate the reduced error rate with KAPA HiFi polymerase and the higher error rate in fragment covered by reverse reads. **b)** Comparison of error rate per base position with HotStarTaq and KAPA HiFi enzymes. A linear regression model explains the relationship between the two variables. Coefficient of determination (R^2^) of 0.73

Finally, the error rate distribution and variation along the sequences was studied (Fig. 1a). The error rate per base position showed high variation with both enzymes, i.e. KAPA HiFi (CV = 74.9%) and HotStarTaq (64.2%). Moreover, sequence information on reverse reads showed substantially higher error rates when compared with sequence information on forward reads. The comparison of the error rate per position with both polymerases showed a significant correlation (*r* = 0.86, *P* < 0.05) with a linear regression model explaining 73% of the variability, meaning an alike error frequency per base pattern with both polymerases suggesting that error distribution is mostly polymerase independent (Fig. 1b). In conclusion, the advantages of performing NGS with the chosen proof-reading DNA polymerase were confirmed and therefore, from this point onward KAPA HiFi DNA polymerase was used to generate *Legionella* libraries.

### *In silico* and *in situ* evaluation of primer specificity

The primer pair Lgsp17F/Lgsp28R was evaluated *in silico* using TestPrime tool against SILVA rRNA gene database (Parc SSU release 123) [26], while allowing none or one mismatch per primer, in order to assess its specificity. The *in silico* analysis showed a good coverage of 84% of the species in the targeted genus*.* Considerable amplification of bacteria belonging to related genus *Methylococcus* (51%) could be expected. Other taxonomic groups, i.e. *Steroidobacter*, *Methylocaldum*, *Aquicella*, *Ricketsiella*, were covered by the primer studied but at a substantially smaller extent (<25%). The primer pair targets the regions V3 and V4 of the 16S rRNA gene of genus *Legionella*, which are two of the most hypervariable regions within the genus studied (Additional file 1: Figure S3). The fragment amplified comprising 421 bp allows a very good resolution of most of the *Legionella* species, including the most clinically relevant species *L. pneumophila* (Additional file 1: Figure S4). The amplicon presents low intragenomic and intergenomic heterogeneity within species *L. pneumophila* (Additional file 1: Figure S5) allowing confident identification.

*In situ* determination of specificity of the primer pair Lgsp17F/Lgsp28R was performed using NGS libraries of triplicates of 7 water samples (two cold drinking water (samples 1 and 2), two hot drinking water [3, 4] and three cooling tower water samples [5–7]). Across these samples, we obtained a total of 1,738,388 sequences after quality filtering and removal of genera with a single read, with the median number of quality sequences in the sample set being 60,455. The mean relative distribution of *Legionella* sequences per sample was 95.2% ± 10.8%. Additionally, 47 other taxonomic groups, such as genera *Methylococcus* and *Ricketsiella*, were amplified but only 4 accounted for 0.1% or more of the sequences classified. These included unclassified *Gammaproteobacteria* SAR86 clade (4.01% ± 9.53%), genera *Methylocaldum* (0.29% ± 0.63%), *Aquicella* (0.10% ± 0.15%) and *Massilia* (0.10% ± 0.12%). The SAR86 clade was solely identified in the cooling tower water samples while the remaining taxa were detected in more than 80% of the total water samples analysed. In the sample set studied, the highest unspecific amplification was observed in sample 7 and corresponded to 29.14% of the classified sequences, with 25.59% of the sequences affiliated to the SAR86 clade. In all other water samples, the percentage of sequences classified as *Legionella* surpassed the 95% threshold, with the highest value being 99.85% (sample 4). Overall, the *in situ* evaluation of the primer pair Lgsp17F/Lgsp28R demonstrated its high specificity for the assessment of *Legionella* community composition.

### Sensitivity and quantification of *Legionella* species using NGS

**Fig. 2:**
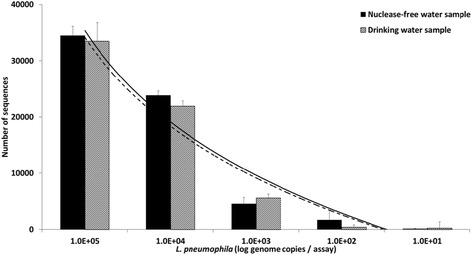
Read abundance relationship with concentration values of *L. pneumophila*. Data retrieved after spike-in of two water samples (nuclease-free water (*black*) and drinking water (hatched)) and graphically represented as bars. A logarithmic regression model elucidates the relationship between the two variables (nuclease free water, y = 3948ln(x) – 14338; drinking water, y = 3824ln(x) – 14063). Logarithmic curves exhibiting the correlation in nuclease-free water (solid line, R^2^ = 0.88) and drinking water (*dashed line*, R^2^ = 0.89) samples are also shown. Y-axis data presented as mean ± SD after analysis of triplicates. Note the x-axis in log scale

To evaluate the sensitivity of the developed NGS approach for the detection of *L. pneumophila* and determine the correlation between sequence abundance and the concentration of the *Legionella* species understudy, triplicates of two samples (nuclease free-water, drinking water) were spiked-in with a dilution range of the targeted bacterium (Fig. 2). In both samples, the minimum concentration of *L. pneumophila* detected was 10 genome copies/assay. The analysis of the sequence abundance along the dilution range of *L. pneumophila* revealed that a logarithmic regression model was the one that best fitted the data obtained for both types of samples. The limit of quantification with this model was shown to be 100 genome copies of *L. pneumophila.* A very good correlation was observed for both nuclease-free water (R^2^ = 0.88) and drinking water (R^2^ = 0.89) spiked samples, suggesting that detection and quantification of *L. pneumophila* is not considerably affected by the bacterial community of the freshwater sample.

**Fig. 3:**
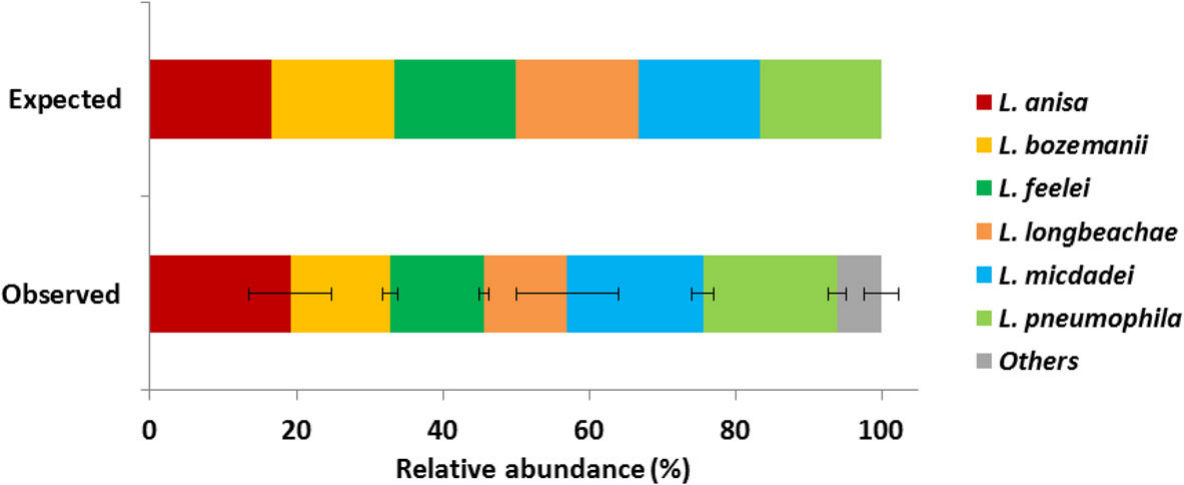
Comparison of expected and observed relative abundances of a mock community after MiSeq sequencing. Data represented as mean ± SD, after analysis of technical replicates (*triplicates*)

A mock community constituted by even concentrations of 16S rDNA of 9 bacterial strains including 6 *Legionella* species was amplified and sequenced in triplicate to determine how accurately the developed NGS assay determines the expected composition (relative read abundances) and diversity of a defined community. A total number of 270,601 16S rRNA gene sequences were analysed, after removal of OTUs with less than 5 reads. All sequences retrieved were affiliated to genus *Legionella*, confirming no amplification of the three non-*Legionella* species. The results showed that the community was not exactly reconstructed, but presented very similar amplification and sequencing of the bacteria analysed (Fig. 3). Amplification of *L. anisa*, *L. micdadei* and *L. pneumophila* was slightly favoured with these bacteria representing 19.2%, 18.5% and 18.4%, respectively, of the expected 16.7% of the total community. The community observed was constituted by on average 93.9% of the expected community. In all triplicates a small percentage of the analysed sequences were affiliated to species not present in the initial community, i.e. *L. dumoffii* (1.8%), *L. santicrucis* (1.7%) and *L. tunisiensis* (0.9%). The presence of *L. dumoffii* and *L. santicrucis* was mostly associated to chimeric and spurious sequences of species *L. longbeachae* and *L. anisa*, whereas the presence of *L. tunisiensis* was linked to erroneous sequences of *L. feelei*. These results confirmed that the methodology is not only highly sensitive, but also to a large extent precise in the amplification of the most relevant *Legionella* species.

### Repeatability of NGS approach for determination of *Legionella* microbiome

Triplicates of the 7 water samples were assessed to determine the technical variation generated by the PCR steps of the *Legionella* libraries preparation using primer pair Lgsp17F/Lgs28R and their subsequent amplification and sequencing. From this dataset, 1,613,470 16S rRNA *Legionella* gene sequences were retrieved with a median number of 76,832 quality sequences per sample classified as *Legionella*. Rarefaction curves for the 21 water samples were plotted (Additional file 1: Figure S6) showing enough sequencing depth as indicated by an average Good’s coverage of 95.3% ± 1.0%.

Alpha-diversity represented by the richness observed and the Shannon’s diversity index (H’) of the individual *Legionella* microbiomes was calculated based on both OTU and phylotype information for all samples after normalisation (Additional file 1: Table S2). Substantial variation in observed OTU richness (OTU_obs_) among replicates was detected with coefficient of variation (CV) values reaching values as high as 36.8% (Sample 4), with an overall mean CV value of 20.8%. In contrast, phylotype richness (PT_obs_) showed a substantially lower variation with an overall CV value of 5.9%. Shannon’s diversity index, based on OTU and phylotype numbers, showed a lower variation among replicates than richness metrics, with an overall mean CV value of 6.5% and 2.9%, respectively. These results seem to be in part due to a significant positive correlation of OTU numbers with sequencing depth (r_s_ = 0.68, P < 0.05), independently of the sample. As OTU-based richness metrics showed lower consistency between replicates, its use is not recommended for *Legionella* microbiome studies with this approach.

**Fig. 4:**
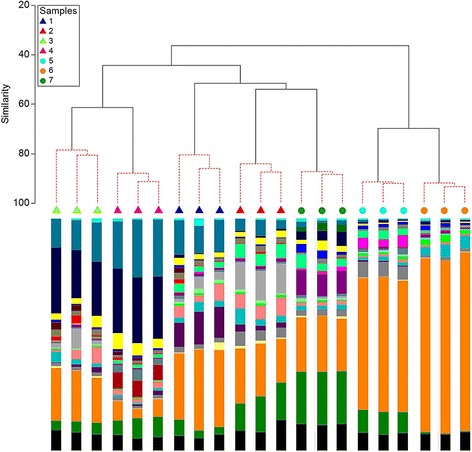
Dendrogram showing group-average hierarchical clustering of triplicates of 7 water samples using Bray-Curtis similarity index. SIMPROF test was performed with 999 permutations. *Red dashed lines* represent samples that do not significantly differ in their *Legionella* community structure (SIMPROF, *P* > 0.05). Technical replicates are represented by the same coloured symbol. The bar graphs represent the abundance of *Legionella* phylotypes. Cold drinking water samples [1, 2]; hot drinking water samples [3, 4] and cooling tower water samples [5–7]

Using Bray-Curtis similarity index, we were able to measure the level of similarity of *Legionella* microbiomes between samples and between replicates within sample. The cluster analysis not only showed significant differences between the water samples studied (ANOSIM *R* = 0.95, *P* < 0.05) but also indicated that the technical variation introduced was not significant in any of the samples (SIMPROF, *P* > 0.05) (Fig. 4). Technical replicates showed, in every case, higher similarity to each other than to a replicate of another sample (Fig. 4 and Additional file 1: Figure S7). Most samples showed very similar community structure between technical replicates (BC > 80), with an overall mean value of 86.8 (Additional file 1: Table S3). This finding was supported by the high Spearman rank correlation coefficient (r_s_) values ranging from 0.79 to 0.93 with a mean of 0.86.

### Profiling of the *Legionella* microbiome in freshwater systems using NGS

**Fig. 5:**
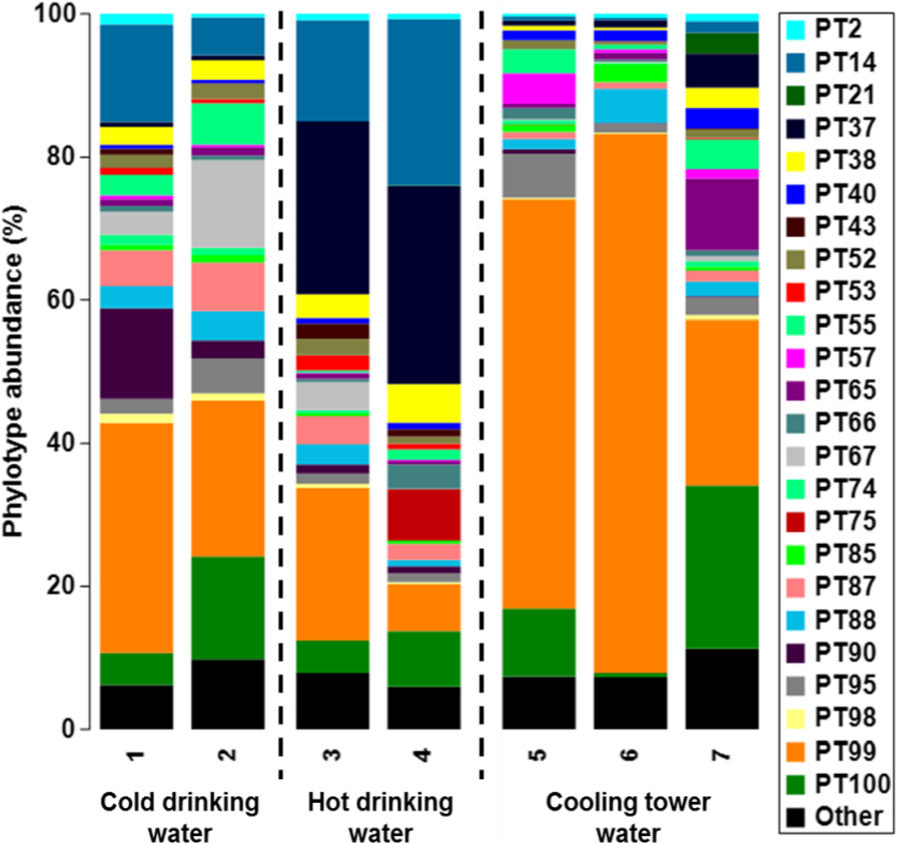
Composition of the *Legionella* microbiome of freshwater samples assessed by 16S rRNA gene sequencing. Mean relative abundance data presented as bar chart show the 24 most abundant phylotypes. Samples from different sampling sites are separated by a dashed line. The phylotypes labelled from 2 to 53 correspond to described *Legionella* species. Identification of all phylotypes detected is given in Additional file 1: Table S4 and their relative abundance is listed in Additional file 1: Table S5

We analysed in more detail the composition and structure of the *Legionella* microbiome of the 7 samples to demonstrate the *in situ* application of the developed NGS approach (Fig. 5). Of the 1,613,470 16S rRNA *Legionella* gene sequences analysed, solely 28.6% were affiliated to a described species, with the abundance of known *Legionella* species presenting minimum values in the cooling tower water samples 5 (8.6%) and 6 (6.1%). In this dataset, 75 *Legionella* phylotypes were detected with the 10 most abundant being PT 99 (34%), PT100 (9.2%), PT37 (8.5%), PT14 (8.4%), PT87 (3.1%), PT67 (2.9%), PT95% (2.8%), PT88 (2.7%), PT55 (2.6%) and PT90 (2.6%) (Additional file 1: Tables S4 and S5). *L. pneumophila* (PT37) and *L. dumoffii* (PT14) were detected in all samples and were among the most abundant phylotypes identified due to their dominance in hot water (samples 3 and 4), representing as much as 26.4% and 23.3%, of the *Legionella* microbiome, respectively. As other potentially pathogenic species, *L. anisa* (PT2), *L. feelei* (PT18)*, L. longbeachae* (PT28)*, L. wadsworthii* (PT50) were observed in the samples, but represented less than 1%. Different samples were clearly separated and replicates were grouped tightly together using a nMDS plot to compare *Legionella* community similarity (Additional file 1: Figure S7). In addition, richness metrics did not reveal substantial difference among samples, showing high phylotype richness among the samples (50 to 58 phylotypes) (Additional file 1: Table S2). Overall, the developed genus-specific approach provided a complete and accurate pathogen profile of all members of the *Legionella* microbiome in a given water sample.

### Comparison between NGS and real-time PCR quantification of *Legionella* microbiome within the bacterial microbiome

**Fig. 6:**
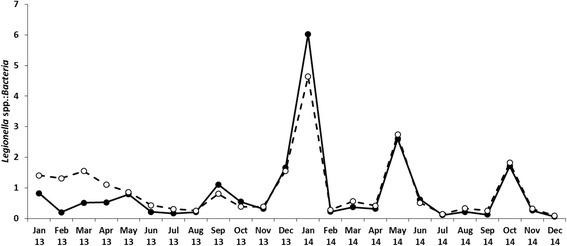
Relative abundances (%) of *Legionella* microbiome within the whole bacterial microbiome in cooling tower water. Determination of abundances was made by NGS (*full circles*) and real-time PCR (open circles). In the NGS-based determination pan-bacterial primers were used and all *Legionella* spp. sequences counted. The real-time abundances were determined by two independent real-time PCR measurements with the pan-bacterial and the genus-specific primer pairs

For a comparison of our NGS approach with real-time PCR in terms of quantification we have chosen to determine the relative abundance of the genus *Legionella* among all *Bacteria* using the same primer pairs for both methodologies. For this purpose a larger set [24] of cooling tower samples was used to determine the relative abundance of all *Legionella* species sequences. These relative abundances for *Legionella* species were also determined by two independent real-time PCR measurements (see Methods) using the same primer pairs as for pan-bacterial and genus-specific NGS library preparation. The two real-time PCR analyses provided the total number of genome units (GU) per assay for all *Legionella* species and all *Bacteria*. These data allowed the calculation of the relative abundance of the *Legionella* microbiome in relation to the whole bacterial microbiome. A comparison of the dynamics of both data sets is given in Fig. 6 and demonstrated very good congruence among both dynamics determined by independent molecular analyses. This observation was supported by a linear regression analysis of the data with a correlation coefficient r^2^ of 0.88. Overall, these comparisons demonstrated that the NGS profiling approach developed at the genus levels provides a robust relative abundance determination of the genus.

### Detection and quantification of individual *Legionella species* in freshwater by NGS

A comparison between the *Legionella* genus-specific NGS approach (using primers Lgsp17F/Lgsp28R) and the pan-bacterial NGS approach (using primers 519 F/907R) was performed to determine the sensitivity and accuracy of the approaches to detect individual *Legionella* species, using *L. pneumophila* as an example, in the 7 reference samples. When using the *Legionella* genus-specific and the pan-bacterial approaches, the average number of sequences per sample (assessed in triplicates) classified as *Legionella* was 76,832 ± 44,270 and 254 ± 127, respectively.

**Fig. 7:**
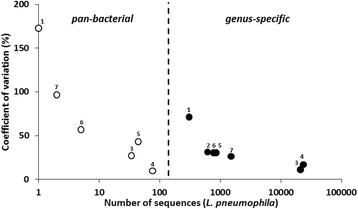
Comparison of the number of *L. pneumophila* sequence reads retrieved by the pan-bacterial (*open circles*) and genus-specific (*filled circles*) NGS approach with the coefficient of variation among triplicates within-sample. Cold drinking water samples [1, 2]; hot drinking water samples [3, 4] and cooling tower water samples [5–7]. A *dashed line* separates the values of both approaches. Please, note that values are not show for samples where *L. pneumophila* was not detected in any of the replicates in the pan-bacterial approach

When applying the *Legionella* genus-specific approach, *L. pneumophila* (PT37) was detected in all replicates of the samples studied. *L. pneumophila* sequence read abundance varied markedly among the samples studied (Fig. 7), with its relative abundance being rather low in the cold drinking water samples (0.47% and 0.68%, respectively) and substantially higher in the hot drinking water samples (24.21% and 27.71%, respectively) (Additional file 1: Table S6). With the pan-bacterial approach, *L. pneumophila* represented, on average, solely 0.03% ± 0.04% of the total bacterial community, with the number of corresponding sequences not surpassing 97 (Fig. 7). Despite the high sequencing depth (Additional file 1: Figure S8), *L. pneumophila* was not detected in all samples. The results demonstrated that in the more specific approach *L. pneumophila* sequence abundance was amplified by two to three orders of magnitude more in terms of read numbers (Fig. 7). This observation can explain the highest sensitivity of the method in cold drinking water and cooling tower water samples where *L. pneumophila* was less abundant. These findings were further corroborated by spike-in experiments that showed that the minimum concentration of *L. pneumophila* detected by the pan-bacterial approach was 10^2^ genome copies/assay, at least one order of magnitude higher than the genus-specific approach (Additional file 1: Figure S9). In addition, the genus specific approach showed a comparatively higher precision of quantification of *L. pneumophila* (CV = 31%) than the pan-bacterial approach (CV = 68%) (Fig. 7). The coefficient of variation negatively correlated with the sequence abundance in both pan-bacterial (r_s_ =−0.94, *P* < 0.05) and genus-specific approaches (r_s_ = −0.99, *P* < 0.05), highlighting the higher difficulty of total community analysis to precisely detect low abundant bacteria. Overall, these comparisons demonstrate that the NGS profiling approach developed at the genus level provided a substantially more sensitive detection and precise quantification of all major *Legionella* species, such *L. pneumophila,* in freshwater samples than the pan-bacterial approach.

## Discussion

### Accuracy of *Legionella* genus-specific NGS approach

NGS technologies have emerged as valuable molecular techniques and are becoming more frequently applied in community studies and pathogen discovery and detection [34]. The accuracy of molecular techniques strongly depends on the agreement between the microorganisms present and their sequences derived from the assay and can be strongly influenced by chimeric artefacts, and amplification and sequencing errors [35, 36]. In NGS, the frequency and type of errors is influenced by the enrichment PCR step and the sequencing platform used. Illumina platforms have been reported to account for the lowest number of sequencing errors and to generate mostly substitutions, whereas in other platforms, such as IonTorrent PGM and Roche 454, indels dominate [37, 38]. To maximise genome-coverage and minimise the error-rate during the library preparation step, the use of high-fidelity DNA polymerases with strong proofreading activity, such as KAPA HiFi, has been recommended [39, 40]. The sequence errors can be measured by two parameters, accuracy that is derived from error rate and quality that is derived from the error-free reads. Our results showed that KAPA HiFi outperformed HotStarTaq in both parameters (Fig. 1). Yet, sequences amplified with both enzymes showed overall a low error rate (<0.5%). Additionally, substitutions were the most common errors supporting and being in the range of previous findings with distinct approaches [37, 38, 41]. With KAPA HiFi DNA polymerase, transition substitutions, which are mostly associated with PCR amplification [42], showed a significant reduction of 46%. This was particularly noticed in substitutions A to G and T to C, which tend to predominate with low fidelity enzymes [43, 44]. Nonetheless, transversions represented a substantial part of the sequencing errors detected which have been reported to be caused mainly by Illumina deep sequencing [41, 45]. These results associated with other findings, such as higher error rate at the end of the reads and in reverse reads confirm that the Illumina sequencing step is responsible for an important portion of the errors observed. Additionally, common error hotspots and systematic errors were found with both enzymes supporting the described existence of DNA sequence and motif-dependent errors [41, 46]. Overall, our results demonstrated the beneficial effect of choosing a high-proofreading enzyme during NGS library preparation.

### Specificity and repeatability of the *Legionella* genus-specific NGS approach

When designing a molecular approach, *in silico* evaluation comprising the target gene to address and the primer pair to choose is of unequivocal importance. The *in silico* analysis of primer pair Lgsp17F/Lgsp28R showed not only good coverage and resolution to the species-level of the bacteria englobed in the genus *Legionella,* but also good specificity. Though *in silico* predictive analysis is of great help, empirical *in situ* analysis should be performed as results may differ considerably [47]. The *in situ* analysis revealed high specificity of the primer pair Lgsp17F/Lgsp28R supporting previous findings [48]. Moreover, the primer pair showed good agreement with *in silico* data, with most of the expected taxa being amplified. The main exception was amplification of bacteria in clade SAR86, which is constituted of uncultured bacteria mostly described in marine environments [49]. Our results suggest that these bacteria can also be abundant in man-made freshwater environments, especially in cooling towers. In general, the primer pair tested showed high specificity (mostly >95%) and provided adequate coverage and sequencing depth of the targeted *Legionella* species.

In NGS studies, technical variability is often introduced during sample preparation, library preparation and sequencing [50]. Although resources tend to be more directed to the use of biological replicates with technical variability being occasionally assessed, measurement and proper estimation of technical variability is of critical importance for reliable data interpretation [50, 51]. Our results for the developed NGS approach demonstrated that technical variability as a result of PCR and/or sequencing stochasticity is undisputable in all water samples but it did not significantly modify the *Legionella* microbiome structure and composition (Fig. 4). Overall, we would draw the same beta-diversity conclusions if no technical replicates were used. These results are in accordance with previous studies using distinct approaches with Illumina technology [52, 53].

### Precision and sensitivity of the *Legionella* genus-specific NGS approach

In PCR-based studies the abundance of microbial taxa is often misrepresented and under-detected due to poor primer coverage and reduced amplification efficiency, inadequate sequencing depth, the presence of spurious amplification and sequencing artefacts, plus incorrect sequence clustering and classification [36, 54]. Our results of the mock community revealed that the approach does not seem to strongly favour the amplification of a particular *Legionella* species (Fig. 3). Yet, our findings give us hints that *Legionella* diversity might be slightly overestimated and that taxonomic assignment might be to a small degree wrongly inferred due to possibly incorrect sequences and/or wrong clustering/classification. The spike-in experiments suggest not only high sensitivity of the approach for detection of *L. pneumophila* but also that sequence abundance is a good indicator of the concentration of the targeted bacteria, allowing precise absolute quantification of *L. pneumophila* abundance in freshwater environments (Fig. 2). The relative abundance of the *Legionella* microbiome among the whole bacterial microbiome was determined independently with two real-time measurements using the same primer pairs as for the NGS methods (Fig. 6). This comparison showed good correspondence and correlation between both independent abundance determinations and demonstrated the reliability of the quantification of the genus-specific NGS approach.

In addition, when comparing the developed *Legionella* genus-specific approach with a broader pan-bacterial approach for the detection of *L. pneumophila* in freshwater samples, a higher detection and more precise quantitative measurement of *L. pneumophila* with the *Legionella* genus-specific approach was shown (Fig. 7 and Additional file 1: Figure S9). A study by Vierheilig et al. [55], using 454 pyrosequencing, failed to detect faecal indicator *E. coli* in wastewater, though it was identified by cultivation. These concerns over the capability of a pan-bacterial approach to detect and exactly quantify low-abundant bacteria seem to be confirmed by our results, despite applying a substantially higher sequencing effort with Illumina MiSeq platform. These findings demonstrated that the developed genus-specific assay is a more adequate tool for sensitive detection and quantification of the targeted *Legionella* species.

### Characterisation of *Legionella* microbiome to the species level by NGS

We applied the *Legionella* genus-specific NGS approach to characterise the *Legionella* microbiome diversity and composition of seven representative freshwater samples. Our results revealed that a major percentage of the 16S rRNA sequences analysed (about 71%) were not affiliated to any described species, demonstrating the importance of molecular tools such as NGS to more accurately measure true *Legionella* diversity and abundances. The analysis of the composition of 7 *Legionella* communities revealed the presence of 75 phylotypes, demonstrating the great *Legionella* richness in freshwater environments, which should be more thoroughly studied (Fig. 5). The breakdown of *Legionella* community composition showed that *L. pneumophila* was the most abundant species and the most abundant potentially pathogenic species in the community, especially in hot water. These results corroborate the single-strand conformation polymorphism (SSCP) fingerprint analyses of cold and hot drinking water by Lesnik et al. [48] but revealed a substantially lower taxonomic resolution of this community profiling technique. We obtained 75 *Legionella* phylotypes whereas the SSCP fingerprints using the same primers only found half the number of phylotypes in almost five times more samples. These findings underline the public health relevance of *L. pneumophila* and justify its attentive monitoring in man-made freshwater systems. In addition, the data presented are indicative of a considerable spatial and temporal variation of the *Legionella* microbiome, highlighting the need of a broader and more detailed study of *Legionella* species at distinct sites and throughout time in order to better comprehend their dynamics, distribution and the factors that might trigger their growth, persistence and dominance.

## Conclusions

In summary, the developed *Legionella* genus-specific Illumina-based approach provides an accurate and reliable detection, quantification and unambiguous taxonomic classification of the *Legionella* microbiome comprising all major *Legionella* species. This assay represents an improvement, in terms of sensitivity and precision, to the widely used pan-bacterial approaches and has the potential to be applied to water quality monitoring of *Legionella* species, most importantly *L. pneumophila*. In addition, it will be a valuable molecular tool in water research to augment the scientific understanding of the *Legionella* species dynamics and the biotic and abiotic drivers impacting *Legionella* community structure and diversity. In general, the developed genus-specific NGS approach should be applicable to other environmentally or medically relevant genera and opens up a new window to look in greater detail into specific parts of the microbiome, especially the patho-biome.

## Additional files

Additional file 1: The additional file provides supplementary material contained in Figures S1 to S9 and Tables S1 to S6. **Figure S1.** Comparison of cumulative 16S rRNA gene V3-V4 sequences abundance with sequence identity to *L. pneumophila* ATCC 33152^T^ using KAPA HiFi and HotStarTaq DNA polymerases. **Figure S2.** Error rate profiling with KAPA HiFi and HotStarTaq DNA polymerases. **Figure S3.** Hypervariable regions within the 16S rRNA gene in the genus *Legionella*. **Figure S4.** Phylogenetic resolution of the 16S rRNA gene V3-V4 region for the genus *Legionella*, amplified by primer pair Lgsp17F/Lgsp28R. **Figure S5.** Sequence identity of *Legionella* 16S rRNA gene V3-V4 sequences to the sequences of *L. pneumophila* ATCC 33152^T^. **Figure S6.** Rarefaction curves of *Legionella* OTUs diversity for 7 water samples using the genus-specific NGS approach. **Figure S7.** Within-sample and inter-sample distinctiveness of *Legionella* microbiome structure. **Figure S8.** Rarefaction curves of bacterial OTUs diversity for 7 water samples using the pan-bacterial NGS approach. **Figure S9.** Sensitive quantitative determination of *L. pneumophila* by the genus-specific and pan-bacterial NGS approach. **Table S1a.** Nucleotide sequences of *Legionella* genus-specific NGS primers, targeting 16S rRNA gene, used in the first amplification step (target-specific) of the library preparation for Illumina MiSeq Sequencing. **Table S1b.** Nucleotide sequences of primers, targeting 16S rRNA gene, used in the second amplification step (multiplexing) of the library preparation for Illumina MiSeq Sequencing. **Table S2.** Alpha-diversity of *Legionella* community between replicates (*n* = 3) within each of the 7 water samples analysed. **Table S3.** Bray-Curtis similarity (BC) and Spearman rank correlation (r_s_) of *Legionella* community between replicates (*n* = 3) within each of the 7 water samples analysed. **Table S4.** Taxonomic assignment of 16S rRNA gene sequences affiliated to genus *Legionella*. **Table S5.** Relative abundance (%) of *Legionella* phylotypes in the 7 freshwater samples analysed. **Table S6.** Quantification of *Legionella* spp. and *L. pneumophila* by NGS. (DOCX 998 kb)
