# Supplementary material for: Development of a genus-specific next generation sequencing approach for sensitive and quantitative determination of the Legionella microbiome in freshwater systems
Source: BMC Microbiol. 2017 Mar 31;17:79. doi: 10.1186/s12866-017-0987-5 (PMC5374610; doi:10.1186/s12866-017-0987-5)
Supplement: Supplementary file 1 — The additional file provides supplementary material contained in Figures S1 to S9 and Tables S1 to S6. Figure S1. Comparison of cumulative 16S rRNA gene V3-V4 sequences abundance with sequence identity to L. pneumophila ATCC 33152T using KAPA HiFi and HotStarTaq DNA polymerases. Figure S2. Error rate profiling with KAPA HiFi and HotStarTaq DNA polymerases. Figure S3. Hypervariable regions within the 16S rRNA gene in the genus Legionella. Figure S4. Phylogenetic resolution of the 16S rRNA gene V3-V4 region for the genus Legionella, amplified by primer pair Lgsp17F/Lgsp28R. Figure S5. Sequence identity of Legionella 16S rRNA gene V3-V4 sequences to the sequences of L. pneumophila ATCC 33152T. Figure S6. Rarefaction curves of Legionella OTUs diversity for 7 water samples using the genus-specific NGS approach. Figure S7. Within-sample and inter-sample distinctiveness of Legionella microbiome structure. Figure S8. Rarefaction curves of bacterial OTUs diversity for 7 water samples using the pan-bacterial NGS approach. Figure S9. Sensitive quantitative determination of L. pneumophila by the genus-specific and pan-bacterial NGS approach. Table S1a. Nucleotide sequences of Legionella genus-specific NGS primers, targeting 16S rRNA gene, used in the first amplification step (target-specific) of the library preparation for Illumina MiSeq Sequencing. Table S1b. Nucleotide sequences of primers, targeting 16S rRNA gene, used in the second amplification step (multiplexing) of the library preparation for Illumina MiSeq Sequencing. Table S2. Alpha-diversity of Legionella community between replicates (n = 3) within each of the 7 water samples analysed. Table S3. Bray-Curtis similarity (BC) and Spearman rank correlation (rs) of Legionella community between replicates (n = 3) within each of the 7 water samples analysed. Table S4. Taxonomic assignment of 16S rRNA gene sequences affiliated to genus Legionella. Table S5. Relative abundance (%) of Legionella phylotypes in the 7 freshwater s [file 12866_2017_987_MOESM1_ESM.docx]

**Development of a genus-specific next generation sequencing approach for sensitive and quantitative determination of the *Legionella* microbiome in freshwater systems**

Additional file 1

Rui P. A. Pereira^a^, Jörg Peplies^b^, Ingrid Brettar^a^, and Manfred G. Höfle^a^ *

^a^ Department of Vaccinology and Applied Microbiology, RG Microbial Diagnostics, Helmholtz Centre for Infection Research (HZI), Inhoffenstr. 7, 38124 Braunschweig, Germany

^b^ Ribocon GmbH, Fahrenheitstraße 1, 28359 Bremen, Germany

*Correspondence: [manfred.hoefle@helmholtz-hzi.de](mailto:manfred.hoefle@helmholtz-hzi.de)

Rui P. A. Pereira, Email: [Rui.Pereira@helmholtz-hzi.de](mailto:Rui.Pereira@helmholtz-hzi.de)

Jörg Peplies Email: [jpeplies@ribocon.com](mailto:jpeplies@ribocon.com)

Ingrid Brettar Email: [Ingrid.Brettar@helmholtz-hzi.de](mailto:Ingrid.Brettar@helmholtz-hzi.de)


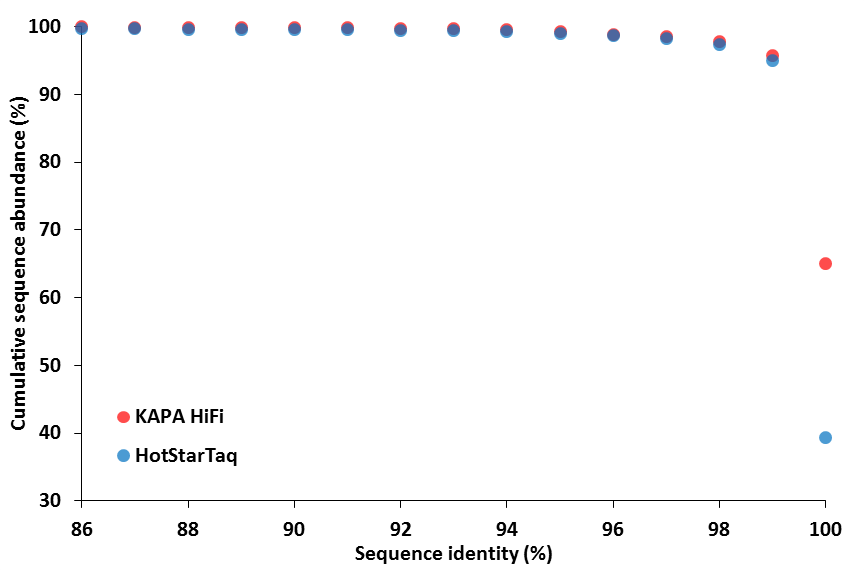


**Figure S1.** **Comparison of cumulative 16S rRNA gene V3-V4 sequences abundance with sequence identity to *L. pneumophila* ATCC 33152^T^ using KAPA HiFi (red circles) and HotStarTaq (blue circles) DNA polymerases.** Data shown as combined analysis of three technical replicates amplified with each enzyme. 65.1% and 39.3% of the total number of sequences retrieved, after amplification with KAPA HiFi and HotStarTaq enzymes, respectively, showed 100% sequence identity. With a sequence identity threshold of 97%, for both enzymes, >98% of the sequences would be assigned to *L. pneumophila*.


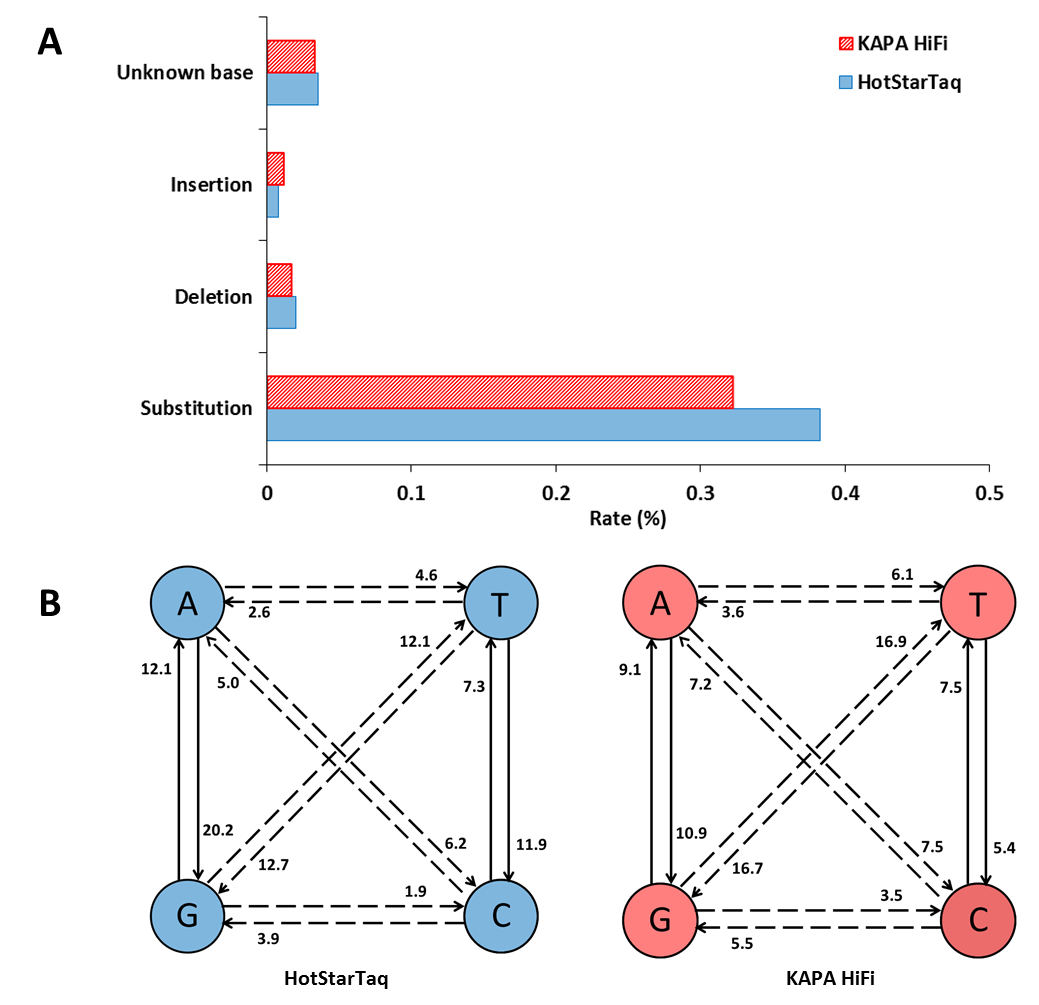


**Figure S2. Error rate profiling with KAPA HiFi and HotStarTaq DNA polymerases.** Analysis of the library preparation and Illumina MiSeq amplification and sequencing using 15,000 reads of *L. pneumophila*. **A)** Bars represent the average rate per base. **B)** Diagram represents the different substitution error profiles as percentage of the total substitutions for HotStarTaq (blue) and KAPA HiFi (red) DNA polymerases. Continuous arrows indicate transitions and dashed arrows indicate transversions.


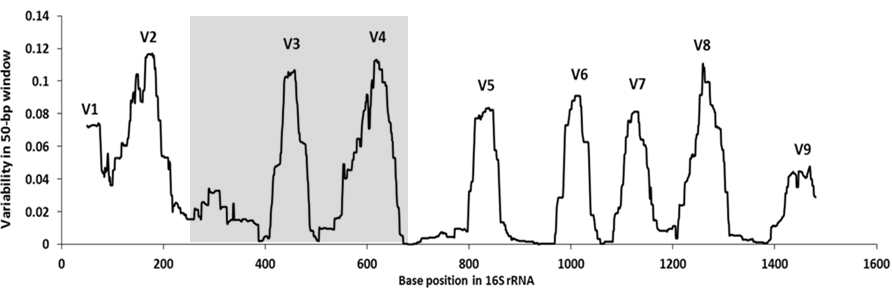


**Figure S3.** **Hypervariable regions within the 16S rRNA gene in the genus *Legionella*.** The graph reflects fluctuations in variability amongst aligned full-length 16S rRNA gene sequences of *Legionella* type strains available in the databases. Variability for each base position was calculated as one minus the frequency of the most common nucleotide. The frequency distribution was then smoothed by taking the mean frequency within a 50-base sliding position. Peaks in variability amongst the aligned sequences represent the 9 hypervariable regions of the 16S rRNA gene in genus *Legionella* (V1-V9). The 16S rRNA gene region amplified by primer pair Lgsp17F/Lgsp28R is highlighted in grey.


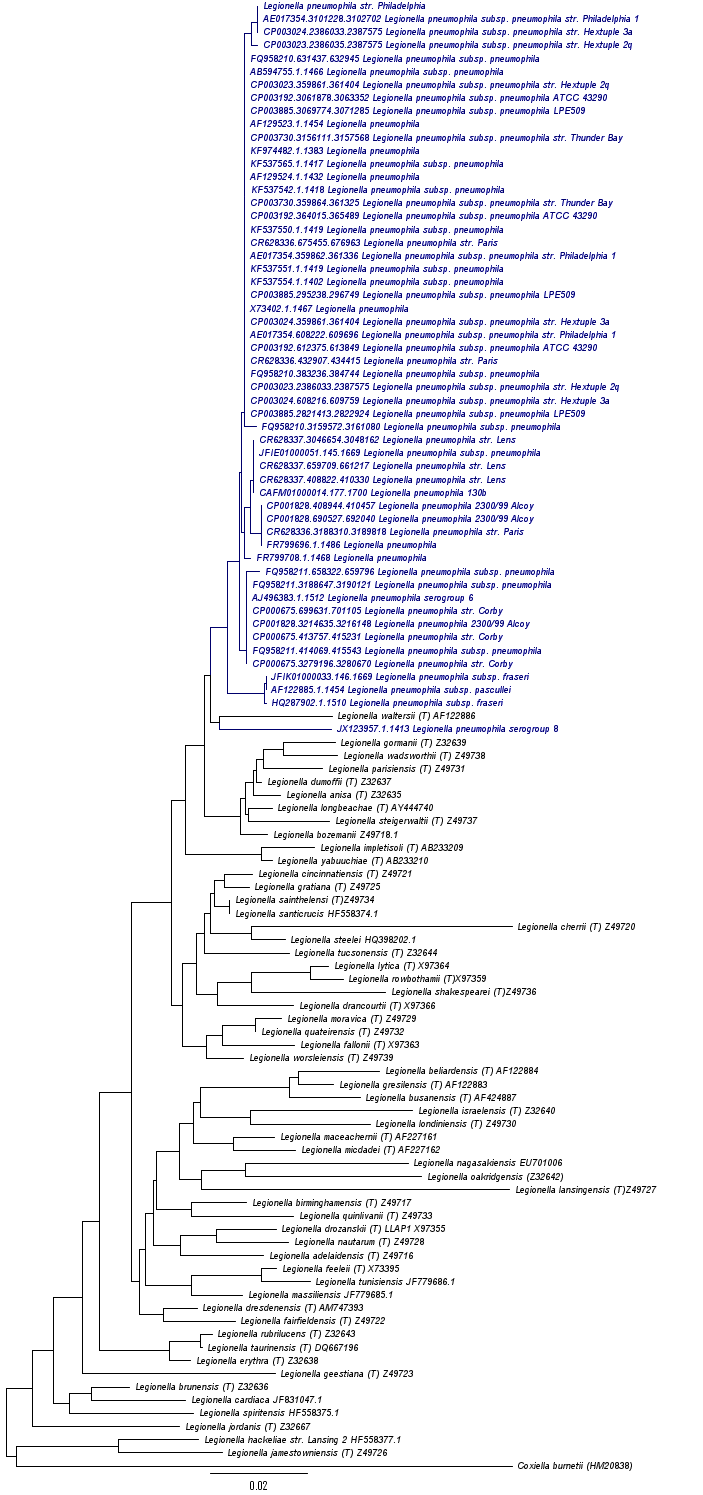


**Figure S4. Phylogenetic resolution of the 16S rRNA gene V3-V4 region for the genus *Legionella*, amplified by primer pair Lgsp17F/Lgsp28R.** Phylogenetic tree shown as a graphic representation generated by the neighbor-joining method from a distance matrix calculated using the Kimura-2 parameter model. *L. pneumophila* 16S rRNA gene sequences retrieved from the non-redundant version of the SILVA SSU 115 NR dataset are shown in blue. A clear cluster composed of *L. pneumophila* 16S rRNA gene fragments is observed confirming the low intragenomic and intergenomic heterogeneity within the species. Please note that the represented branch length scale corresponds to 2 nucleotide substitutions per 100 nucleotide positions.

**
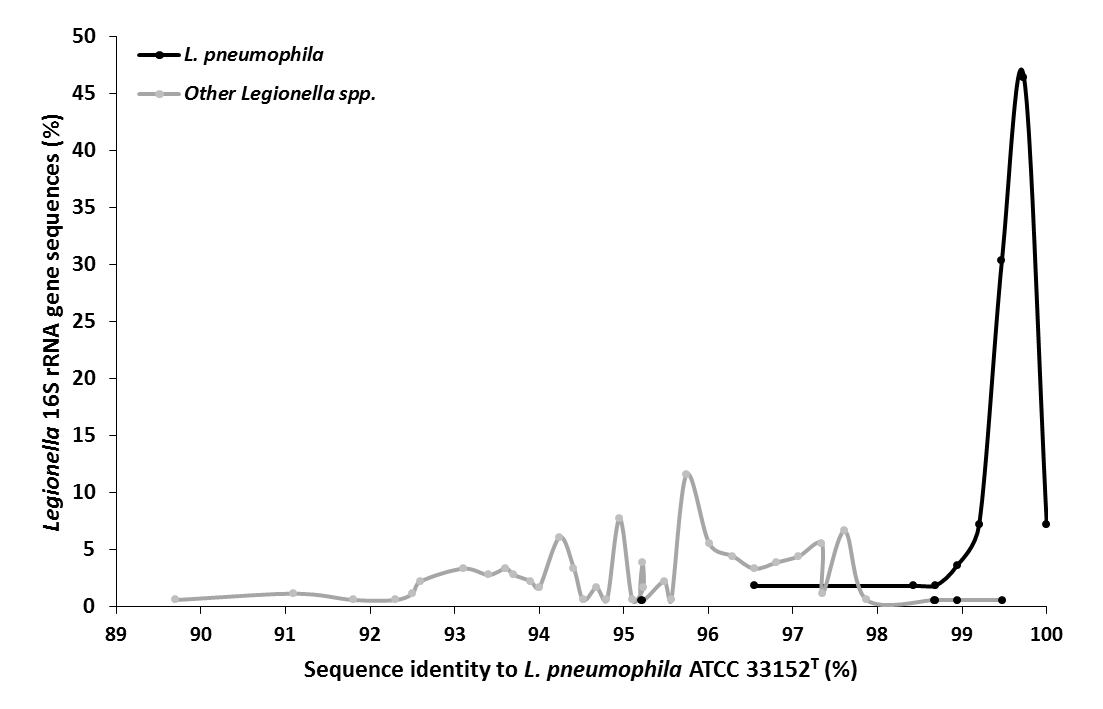
**

**Figure S5. Sequence identity of *Legionella* 16S rRNA gene V3-V4 sequences to the sequences of *L. pneumophila* ATCC 33152^T^.** 56 and 182 16S rRNA gene sequences classified as *L. pneumophila* (black line) and as *Legionella* species other than *L. pneumophila* (grey line), respectively were retrieved from the non-redundant version of the SILVA SSU 115 NR dataset. Black circles indicate V3-V4 16S fragments with the highest sequence identity to *L. pneumophila*. 47 *L. pneumophila* sequences (83.9%) showed sequence identity ≥99.5% to the type strain. Of the 182 16S rRNA gene sequences classified to a *Legionella* species other than *L. pneumophila* analysed only 5 (2.8%) V3-V4 16S fragments showed higher sequence identity to *L. pneumophila* than to the expected species.


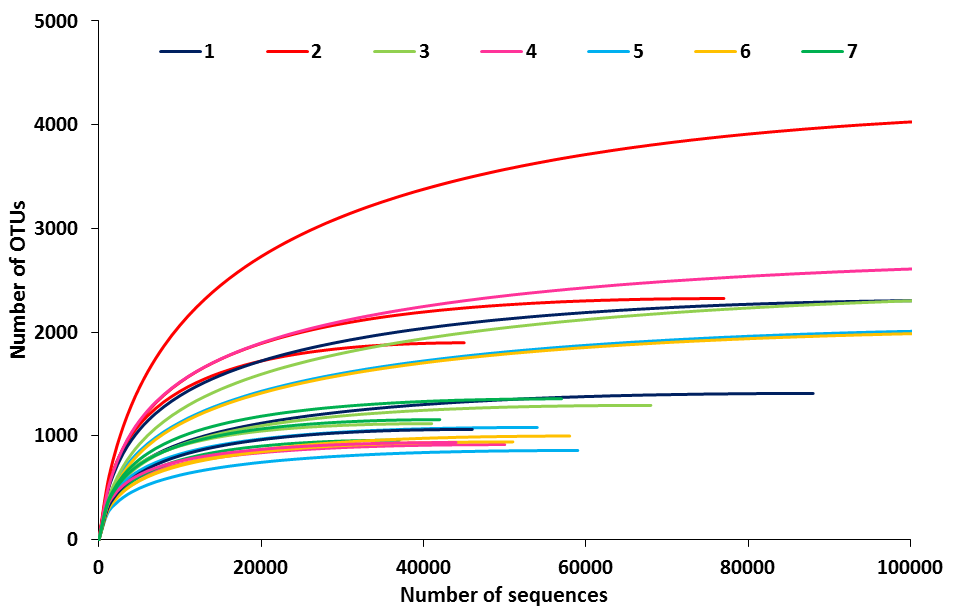


**Figure S6. Rarefaction curves of *Legionella* OTUs diversity for 7 water samples using the genus-specific NGS approach.** Each water sample comprises three technical replicates separately analysed.

**
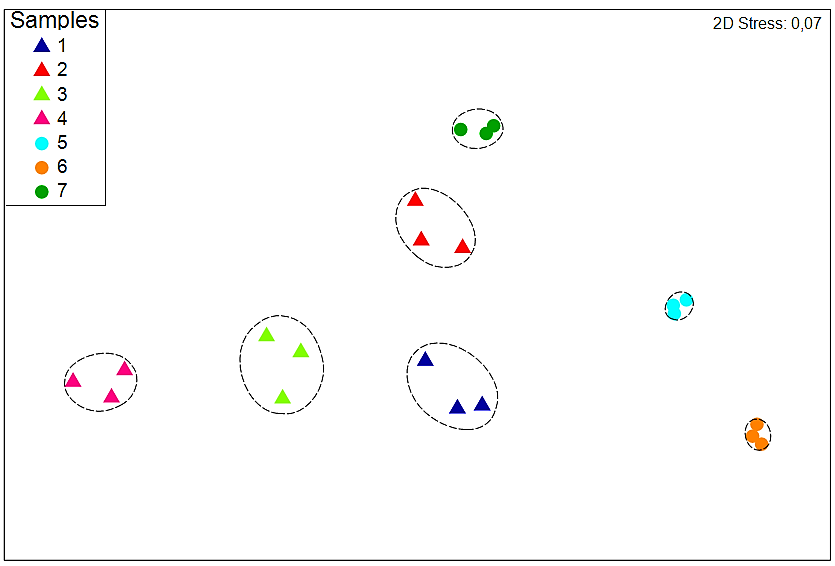
**

**Figure S7. Within-sample and inter-sample distinctiveness of *Legionella* microbiome structure.** Non-metric multi-dimensional scaling (nMDS) plot depicting *Legionella* community composition of technical replicates (n = 3) of 7 water samples. Data are untransformed and based on Bray-Curtis similarity calculation. Samples comprised in area delimited by dashed lines do not show statistically significant difference in their *Legionella* community (SIMPROF, P>0.05). Technical replicates are represented by symbols of the same colour. Cold drinking water samples (1,2); hot drinking water samples (3,4); cooling tower water samples (5-7). A 2D stress value of 0.07 indicates a good fit of the data.


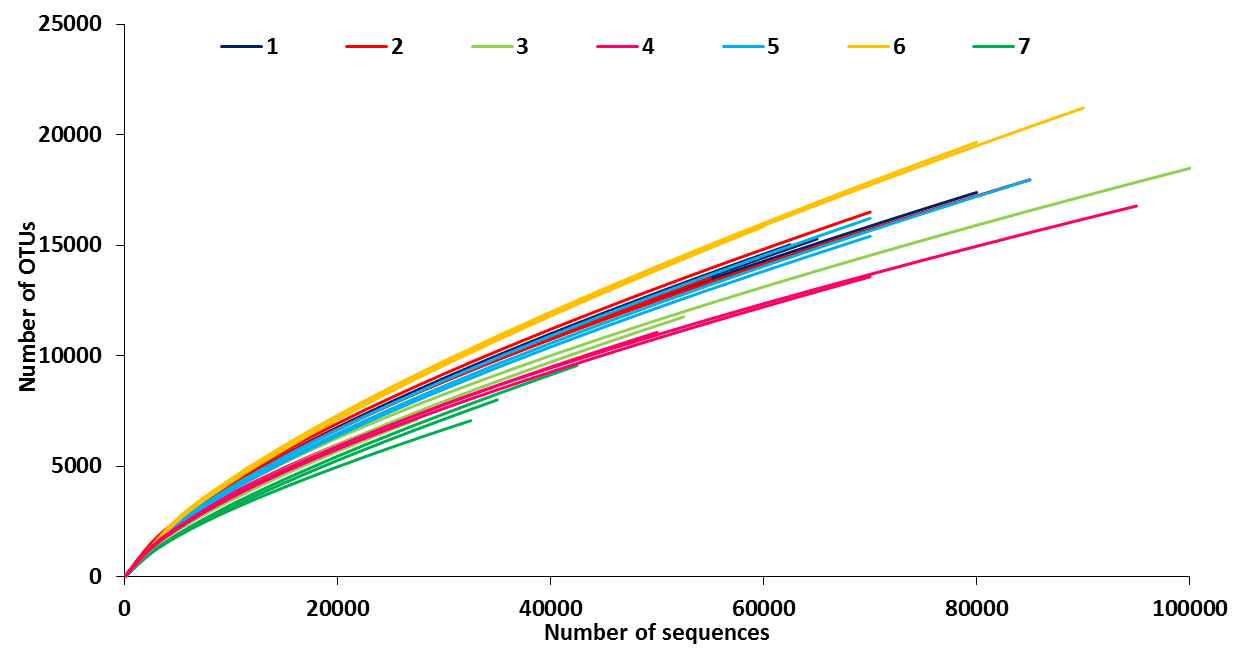


**Figure S8. Rarefaction curves of bacterial OTUs diversity for 7 water samples using the pan-bacterial NGS approach.** Each water sample comprises three technical replicates separately analysed.


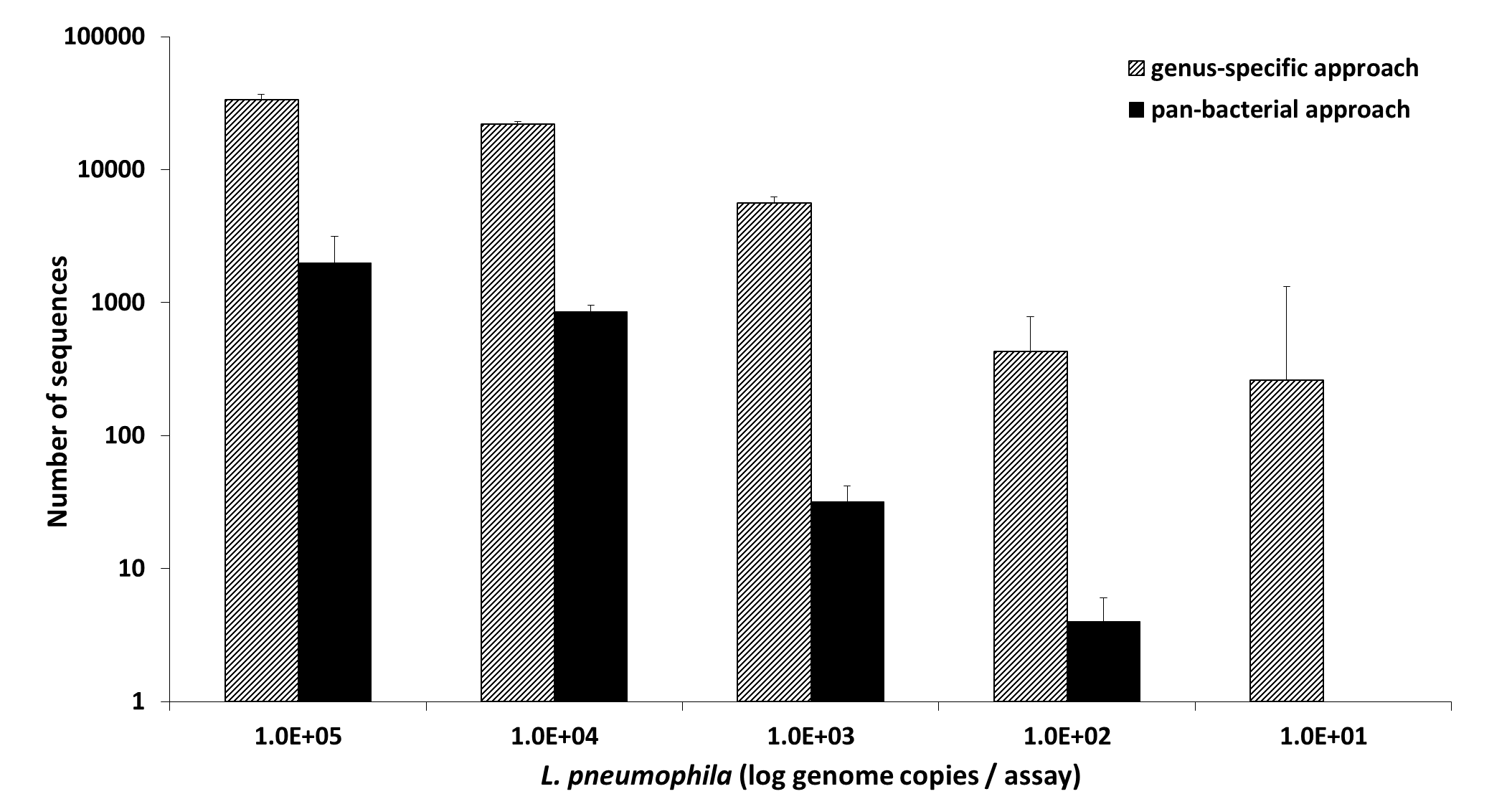


**Figure S9. Sensitive quantitative determination of *L. pneumophila* by the genus-specific (hatched) and pan-bacterial (black) NGS approaches.** Data retrieved after spike-in of a drinking water sample with a dilution range of *L. pneumophila* (10^1^ to 10^5^ genome copies) and graphically represented as bars. Note both x and y axes in log scale.

**Table S1A. Nucleotide sequences of *Legionella* genus-specific NGS primers, targeting 16S rRNA gene, used in the first amplification step (target-specific) of the library preparation for Illumina MiSeq Sequencing.** Italic lowercase are binding sites for the Illumina sequencing primers. Bold uppercase letters highlight the barcode/index sequence. Italic uppercase highlights the linker which links the barcode with the *Legionella* 16S rRNA gene complementary primer sequence.

| **Forward Primers** | **Sequence (5’ to 3’)** |
| --- | --- |
| **F1** | *acactctttccctacacgacgctcttccgatct* **AATGGT** *CA* GGCCTACCAAGGCGACGATCG |
| **F2** | *acactctttccctacacgacgctcttccgatct* **ATTCTC** *CA* GGCCTACCAAGGCGACGATCG |
| **F3** | *acactctttccctacacgacgctcttccgatct* **ATACCT** *CA* GGCCTACCAAGGCGACGATCG |
| **F4** | *acactctttccctacacgacgctcttccgatct* **AATCCA** *CA* GGCCTACCAAGGCGACGATCG |
| **F5** | *acactctttccctacacgacgctcttccgatct* **ATTGAG** *CA* GGCCTACCAAGGCGACGATCG |
| **F6** | *acactctttccctacacgacgctcttccgatct* **CCTTGA** *CA* GGCCTACCAAGGCGACGATCG |
| **F7** | *acactctttccctacacgacgctcttccgatct* **CCGTAG** *CA* GGCCTACCAAGGCGACGATCG |
| **F8** | *acactctttccctacacgacgctcttccgatct* **CGGAAC** *CA* GGCCTACCAAGGCGACGATCG |
| **F9** | *acactctttccctacacgacgctcttccgatct* **CGATTA** *CA* GGCCTACCAAGGCGACGATCG |
| **F10** | *acactctttccctacacgacgctcttccgatct* **CACATA** *CA* GGCCTACCAAGGCGACGATCG |
| **F11** | *acactctttccctacacgacgctcttccgatct* **GAATCT** *CA* GGCCTACCAAGGCGACGATCG |
| **F12** | *acactctttccctacacgacgctcttccgatct* **GATAAG** *CA* GGCCTACCAAGGCGACGATCG |
| **F13** | *acactctttccctacacgacgctcttccgatct* **GGATGC** *CA* GGCCTACCAAGGCGACGATCG |
| **F14** | *acactctttccctacacgacgctcttccgatct* **GAACGG** *CA* GGCCTACCAAGGCGACGATCG |
| **F15** | *acactctttccctacacgacgctcttccgatct* **GGACTT** *CA* GGCCTACCAAGGCGACGATCG |
| **F16** | *acactctttccctacacgacgctcttccgatct* **TGAGGA** *CA* GGCCTACCAAGGCGACGATCG |
| **F17** | *acactctttccctacacgacgctcttccgatct* **TACCCA** *CA* GGCCTACCAAGGCGACGATCG |
| **F18** | *acactctttccctacacgacgctcttccgatct* **TTCAAC** *CA* GGCCTACCAAGGCGACGATCG |
| **F19** | *acactctttccctacacgacgctcttccgatct* **TCATGT** *CA* GGCCTACCAAGGCGACGATCG |
| **F20** | *acactctttccctacacgacgctcttccgatct* **TCGCTT** *CA* GGCCTACCAAGGCGACGATCG |
| **Reverse Primer** | **Sequence (5’ to 3’)** |
| **R0** | *gtgactggagttcagacgtgtgctcttccgatct* CACCGGAAATTCCACTACCCTCTC |

**Table S1B. Nucleotide sequences of primers, targeting 16S rRNA gene, used in the second amplification step (multiplexing) of the library preparation for Illumina MiSeq Sequencing.** Underlined lowercase letters are binding sites for the Illumina’s flow cell and italic lowercase are binding sites for the Illumina sequencing primers. Bold uppercase letters highlight the index sequence.

| **Reverse Primers** | **Sequence (5’ to 3’)** |
| --- | --- |
| **IDX_R1** | caagcagaagacggcatacgagat **CGTGAT** *gtgactggagttcagacgtgtgctcttccgatct* |
| **IDX_R2** | caagcagaagacggcatacgagat **ACATCG** *gtgactggagttcagacgtgtgctcttccgatct* |
| **IDX_R3** | caagcagaagacggcatacgagat **GCCTAA** *gtgactggagttcagacgtgtgctcttccgatct* |
| **IDX_R4** | caagcagaagacggcatacgagat **TGGTCA** *gtgactggagttcagacgtgtgctcttccgatct* |
| **IDX_R5** | caagcagaagacggcatacgagat **CACTGT** *gtgactggagttcagacgtgtgctcttccgatct* |
| **IDX_R6** | caagcagaagacggcatacgagat **ATTGGC** *gtgactggagttcagacgtgtgctcttccgatct* |
| **IDX_R7** | caagcagaagacggcatacgagat **GATCTG** *gtgactggagttcagacgtgtgctcttccgatct* |
| **IDX_R8** | caagcagaagacggcatacgagat **TCAAGT** *gtgactggagttcagacgtgtgctcttccgatct* |
| **IDX_R9** | caagcagaagacggcatacgagat **CTGATC** *gtgactggagttcagacgtgtgctcttccgatct* |
| **IDX_R10** | caagcagaagacggcatacgagat **AAGCTA** *gtgactggagttcagacgtgtgctcttccgatct* |
| **IDX_R11** | caagcagaagacggcatacgagat **GTAGGC** *gtgactggagttcagacgtgtgctcttccgatct* |
| **IDX_R12** | caagcagaagacggcatacgagat **TACAAG** *gtgactggagttcagacgtgtgctcttccgatct* |
| **Forward primer** |  |
| **Illu_Mplex** | aatgatacggcgaccaccgagatct *acactctttccctacacgacgctcttccgatct* |

**Table S2. Alpha-diversity of *Legionella* community between replicates (n = 3) within each of the 7 water samples analysed.** Observed richness and Shannon’s diversity Index (*H’*) calculated with OTU and phylotype numbers are listed.

| **Sample** | **OTU_obs_** | ***H'* (OTU)** | **PT_obs_** | ***H’* (PT)** |
| --- | --- | --- | --- | --- |
| 1 | 1383 ± 334 | 7.57 ± 0.89 | 51 ± 2 | 4.00 ± 0.13 |
| 2 | 2141 ± 363 | 8.98 ± 0.28 | 58 ± 5 | 4.24 ± 0.10 |
| 3 | 1345 ± 201 | 7.66 ± 0.55 | 55 ± 2 | 3.82 ± 0.15 |
| 4 | 1296 ± 476 | 7.89 ± 0.54 | 50 ± 3 | 3.61 ± 0.09 |
| 5 | 1159 ± 281 | 7.19 ± 0.57 | 52 ± 5 | 3.14 ± 0.12 |
| 6 | 1153 ± 240 | 6.69 ± 0.18 | 57 ± 2 | 2.18 ± 0.08 |
| 7 | 1363 ± 107 | 7.48 ± 0.43 | 57 ± 3 | 4.21 ± 0.04 |

**Table S3. Bray-Curtis similarity (BC) and Spearman rank correlation (r_s_) of *Legionella* community between replicates (n = 3) within each of the 7 water samples analysed.** Values shown as mean ± SD. BC coefficient values are bound between 0 and 100. Spearman rank correlation r_s_ mean values are bound between -1 and 1.

| **Sample** | **Type of water** | **BC** | **r_s_** |
| --- | --- | --- | --- |
| 1 | cold drinking | 82.28 ± 3.63 | 0.88 ± 0.01 |
| 2 | cold drinking | 85.27 ± 2.51 | 0.93 ± 0.02 |
| 3 | hot drinking | 79.13 ± 1.65 | 0.79 ± 0.01 |
| 4 | hot drinking | 89.07 ± 2.03 | 0.89 ± 0.01 |
| 5 | cooling tower | 91.61 ± 0.40 | 0.85 ± 0.01 |
| 6 | cooling tower | 92.34 ± 0.85 | 0.79 ± 0.05 |
| 7 | cooling tower | 87.65 ± 0.69 | 0.89 ± 0.02 |

**Table S4. Taxonomic assignment of 16S rRNA gene sequences affiliated to genus *Legionella*.**

| **PT** | **Sequence identity** |  | **PT** | **Sequence identity** |
| --- | --- | --- | --- | --- |
| **PT1** | ≥97% *L. adelaidensis* |  | **PT58** | 93-96% *L. bozemannii* |
| **PT2** | ≥97% *L. anisa* |  | **PT59** | 93-96% *L. brunensis* |
| **PT4** | ≥97% *L. birminghamensis* |  | **PT60** | 93-96% *L. busanensis* |
| **PT8** | ≥97% *L. cardiaca* |  | **PT61** | 93-96% *L. cardiaca* |
| **PT10** | ≥97% *L. cincinnatiensis* |  | **PT63** | 93-96% *L. cincinnatiensis* |
| **PT11** | ≥97% *L. drancourtii* |  | **PT64** | 93-96% *L. drancourtii* |
| **PT12** | ≥97% *L. dresdenensis* |  | **PT65** | 93-96% *L. dresdenensis/L. fairfieldensis* |
| **PT13** | ≥97% *L. drozanskii* |  | **PT66** | 93-96% *L. drozanskii* |
| **PT14** | ≥97% *L. dumoffii* |  | **PT67** | 93-96% *L. dumoffii* |
| **PT16** | ≥97% *L. fairfieldensis* |  | **PT69** | 93-96% *L. fallonii* |
| **PT17** | ≥97% *L. fallonii* |  | **PT70** | 93-96% *L. feelei* |
| **PT18** | ≥97% *L. feelei* |  | **PT71** | 93-96% *L. geestiana* |
| **PT19** | ≥97% *L. geestiana* |  | **PT72** | 93-96% *L. gormanii* |
| **PT21** | ≥97% *L. gratiana* |  | **PT73** | 93-96% *L. gratiana* |
| **PT24** | ≥97% *L. impletisoli* |  | **PT74** | 93-96% *L. gresilensis* |
| **PT28** | ≥97% *L. longbeachae* |  | **PT75** | 93-96% *L. hackeliae* |
| **PT31** | ≥97% *L. maceachernii* |  | **PT77** | 93-96% *L. jordanis* |
| **PT32** | ≥97% *L. massiliensis* |  | **PT79** | 93-96% *L. longbeachae* |
| **PT34** | ≥97% *L. nautarum* |  | **PT80** | 93-96% *L. maceachernii* |
| **PT37** | ≥97% *L. pneumophila* |  | **PT81** | 93-96% *L. massiliensis* |
| **PT38** | ≥97% *L. quateirensis* |  | **PT82** | 93-96% *L. micdadei* |
| **PT39** | ≥97% *L. quinlivani* |  | **PT84** | 93-96% *L. nagasakiensis* |
| **PT40** | ≥97% *L. rowbothamii* |  | **PT85** | 93-96% *L. nautarum* |
| **PT43** | ≥97% *L. santicrucis* |  | **PT87** | 93-96% *L. pneumophila* |
| **PT44** | ≥97% *L. shakespearei* |  | **PT88** | 93-96% *L. rowbothamii/L. lytica* |
| **PT45** | ≥97% *L. steelei* |  | **PT90** | 93-96% *L. santicrucis* |
| **PT47** | ≥97% *L. taurinensis* |  | **PT91** | 93-96% *L. shakespearei* |
| **PT48** | ≥97% *L. tucsonensis* |  | **PT92** | 93-96% *L. spiritensis* |
| **PT49** | ≥97% *L. tunisiensis* |  | **PT93** | 93-96% *L. steelei* |
| **PT50** | ≥97% *L. wadsworthii* |  | **PT94** | 93-96% *L. steigerwaltii* |
| **PT51** | ≥97% *L. waltersii* |  | **PT95** | 93-96% *L. tucsonensis* |
| **PT52** | ≥97% *L. worsleiensis* |  | **PT96** | 93-96% *L. tunisiensis* |
| **PT53** | ≥97% *L. yabuuchiae* |  | **PT97** | 93-96% *L. wadsworthii* |
| **PT54** | 93-96% *L. adelaidensis* |  | **PT98** | 93-96% *L. waltersii* |
| **PT55** | 93-96% *L. anisa* |  | **PT99** | 93-96% *L. impletisoli*/*L.yabuuchiae* |
| **PT56** | 93-96% *L. beliardiensis* |  | **PT100** | 93-96% *L. erythra*/ *L. taurinensis*/*L. rubrilucens* |
| **PT57** | 93-96% *L. birminghamensis* |  | **PT101** | 93-96% *L. parisiensis* |

**Table S5. Relative abundance (%) of *Legionella* phylotypes in the 7 freshwater samples analysed.** Mean values after triplicate analysis are listed. n.d, not detected.

| **Phylotype** |  | **Freshwater samples** | | | | | | |
| --- | --- | --- | --- | --- | --- | --- | --- | --- |
|  |  | **1** | **2** | **3** | **4** | **5** | **6** | **7** |
| **PT1** |  | 0.607 | 0.961 | 0.993 | 0.749 | n.d. | 0.014 | 0.036 |
| **PT2** |  | 1.484 | 0.479 | 0.872 | 0.709 | 0.335 | 0.599 | 1.028 |
| **PT4** |  | 0.008 | 0.094 | 0.117 | 0.014 | n.d. | 0.053 | n.d. |
| **PT8** |  | n.d. | n.d. | 0.008 | n.d. | 0.019 | n.d. | n.d. |
| **PT10** |  | n.d. | n.d. | n.d. | n.d. | n.d. | 0.006 | 0.006 |
| **PT11** |  | 0.708 | 0.387 | 0.279 | 0.378 | 0.126 | 0.013 | 0.347 |
| **PT12** |  | 0.099 | 0.111 | 0.124 | 0.011 | 0.448 | 0.068 | 0.367 |
| **PT13** |  | 0.209 | 0.201 | 0.269 | 0.247 | 0.054 | 0.047 | 0.500 |
| **PT14** |  | 13.582 | 5.325 | 14.087 | 23.270 | 0.587 | 0.283 | 1.612 |
| **PT16** |  | n.d. | 0.028 | 0.072 | 0.023 | 0.004 | 0.030 | 0.065 |
| **PT17** |  | n.d. | 0.006 | n.d. | 0.003 | 0.004 | 0.006 | n.d. |
| **PT18** |  | 0.104 | 0.581 | 0.139 | 0.012 | 0.003 | 0.211 | 0.412 |
| **PT19** |  | n.d. | n.d. | 0.005 | n.d. | 0.039 | 0.057 | 0.128 |
| **PT21** |  | 0.011 | 0.019 | 0.041 | 0.061 | n.d. | 0.016 | 2.902 |
| **PT24** |  | 0.023 | 0.050 | 0.037 | 0.041 | n.d. | n.d. | n.d. |
| **PT28** |  | n.d. | 0.076 | 0.018 | 0.129 | 0.009 | 0.018 | n.d. |
| **PT31** |  | n.d. | n.d. | 0.019 | n.d. | n.d. | 0.191 | 1.926 |
| **PT32** |  | 0.040 | 0.024 | 0.006 | 0.007 | 0.012 | 0.230 | n.d. |
| **PT34** |  | n.d. | 0.019 | 0.036 | n.d. | 0.073 | 0.007 | 0.007 |
| **PT37** |  | 0.688 | 0.676 | 24.208 | 27.705 | 0.760 | 1.025 | 4.785 |
| **PT38** |  | 2.473 | 2.695 | 3.314 | 5.371 | 0.610 | 0.334 | 2.835 |
| **PT39** |  | n.d. | 0.024 | 0.005 | n.d. | 0.009 | n.d. | 0.076 |
| **PT40** |  | 0.606 | 0.495 | 0.717 | 0.903 | 1.393 | 1.526 | 2.818 |
| **PT43** |  | 0.848 | 0.121 | 2.146 | 1.050 | 0.025 | 0.107 | 0.158 |
| **PT44** |  | 0.027 | n.d. | 0.061 | n.d. | 1.187 | 0.584 | 0.445 |
| **PT45** |  | 0.169 | 0.019 | 0.051 | n.d. | 0.017 | 0.025 | n.d. |
| **PT47** |  | 0.164 | 0.337 | 0.813 | 1.018 | 0.523 | 0.035 | 0.185 |
| **PT48** |  | 0.074 | 0.355 | 0.435 | 0.292 | 0.225 | 0.074 | 0.928 |
| **PT49** |  | n.d. | n.d. | 0.076 | n.d. | n.d. | n.d. | 0.165 |
| **PT50** |  | n.d. | n.d. | 0.054 | 0.030 | n.d. | n.d. | n.d. |
| **PT51** |  | 0.123 | 0.106 | 1.125 | 0.353 | 0.887 | 0.136 | 0.460 |
| **PT52** |  | 1.803 | 2.099 | 2.303 | 0.995 | 1.248 | 0.384 | 1.140 |
| **PT53** |  | 1.003 | 0.609 | 2.101 | 0.786 | 0.015 | 0.070 | 0.228 |
| **PT54** |  | 1.391 | 1.171 | 0.441 | 0.356 | 0.239 | 0.108 | 0.611 |
| **PT55** |  | 2.849 | 5.697 | 0.346 | 1.480 | 3.342 | 0.608 | 4.197 |
| **PT56** |  | 0.010 | 0.036 | 0.041 | 0.002 | 0.091 | 0.203 | n.d. |
| **PT57** |  | 0.517 | 0.369 | 0.166 | 0.192 | 4.302 | 0.501 | 1.265 |
| **PT58** |  | n.d. | n.d. | n.d. | 0.056 | n.d. | n.d. | n.d. |
| **PT59** |  | 0.062 | 0.125 | 0.019 | n.d. | 0.022 | 0.043 | 0.182 |
| **PT60** |  | 0.013 | n.d. | n.d. | 0.001 | 0.011 | n.d. | n.d. |
| **PT61** |  | 0.016 | 0.035 | 0.043 | 0.004 | 0.002 | 0.199 | 0.098 |
| **PT63** |  | 0.004 | 0.025 | 0.010 | 0.469 | 0.003 | 0.020 | n.d. |
| **PT64** |  | 0.160 | 0.951 | 0.024 | 0.458 | 0.068 | 0.005 | 0.015 |
| **PT65** |  | 1.145 | 1.661 | 0.878 | 0.439 | 1.563 | 1.082 | 10.389 |
| **PT66** |  | 0.838 | 0.656 | 0.446 | 3.423 | 1.510 | 0.319 | 0.843 |
| **PT67** |  | 3.222 | 12.310 | 3.939 | 0.043 | 0.144 | 0.142 | 0.686 |
| **PT69** |  | n.d. | 0.027 | 0.001 | 0.197 | 0.015 | 0.033 | 0.010 |
| **PT70** |  | 0.532 | 1.209 | 0.171 | 0.061 | 0.206 | 1.227 | 0.371 |
| **PT71** |  | 0.041 | 0.012 | 0.077 | n.d. | 0.005 | 0.113 | 0.034 |
| **PT72** |  | n.d. | 0.004 | n.d. | 0.063 | n.d. | n.d. | n.d. |
| **PT73** |  | 0.189 | 0.103 | 0.304 | 0.446 | n.d. | 0.256 | 0.330 |
| **PT74** |  | 1.387 | 1.003 | 0.446 | n.d. | 0.571 | 0.150 | 0.792 |
| **PT75** |  | 0.012 | 0.016 | 0.008 | 7.148 | 0.016 | n.d. | 0.019 |
| **PT77** |  | n.d. | n.d. | 0.015 | 0.004 | n.d. | 0.205 | n.d. |
| **PT79** |  | 0.031 | 0.096 | 0.009 | 0.030 | 0.077 | 0.007 | 0.252 |
| **PT80** |  | 0.049 | 0.025 | 0.004 | n.d. | 0.356 | 0.485 | 0.080 |
| **PT81** |  | 0.210 | 0.192 | 0.024 | 0.053 | 0.205 | 0.162 | 0.066 |
| **PT82** |  | 0.010 | 0.020 | 0.001 | 0.001 | 0.072 | 0.385 | 0.229 |
| **PT84** |  | 0.012 | 0.005 | 0.005 | n.d. | 0.074 | 0.380 | 0.023 |
| **PT85** |  | 0.367 | 0.653 | 0.511 | 0.159 | 0.497 | 0.223 | 0.893 |
| **PT87** |  | 5.068 | 6.765 | 3.931 | 2.250 | 0.964 | 0.915 | 1.547 |
| **PT88** |  | 3.088 | 4.090 | 2.818 | 0.883 | 1.454 | 4.751 | 2.082 |
| **PT90** |  | 12.616 | 2.511 | 1.266 | 1.003 | 0.610 | 0.069 | 0.179 |
| **PT91** |  | 0.005 | 0.033 | 0.013 | 0.008 | 0.477 | 0.100 | 0.079 |
| **PT92** |  | n.d. | 0.006 | 0.004 | 0.098 | 0.038 | 0.703 | 0.294 |
| **PT93** |  | 0.319 | 0.926 | 0.114 | 0.107 | 0.077 | 0.346 | 0.745 |
| **PT95** |  | 2.079 | 4.872 | 1.424 | 1.251 | 6.183 | 1.221 | 2.434 |
| **PT96** |  | 0.185 | 0.073 | 0.026 | 0.031 | 0.182 | 0.136 | 0.526 |
| **PT97** |  | 0.068 | 0.200 | 0.003 | 0.037 | 0.006 | n.d. | 0.026 |
| **PT98** |  | 1.273 | 1.003 | 0.551 | 0.200 | 0.199 | 0.181 | 0.606 |
| **PT99** |  | 32.917 | 22.839 | 21.760 | 7.089 | 58.302 | 78.005 | 23.711 |
| **PT100** |  | 4.460 | 14.373 | 4.541 | 7.801 | 9.507 | 0.567 | 22.828 |
| **PT101** |  | n.d. | 0.009 | 1.085 | n.d. | n.d. | n.d. | n.d. |

**Table S6. Quantification of *Legionella* spp. and *L. pneumophila* by NGS.** Values shown as mean relative abundance ± SD. n.d, not detected.

| **Sample** |  | **Genus-specific approach** |  | **Pan-bacterial approach** | | |
| --- | --- | --- | --- | --- | --- | --- |
|  |  | *L. pneumophila*:*Legionella* spp. |  | *L. pneumophila*:*Legionella* spp. | *L. pneumophila*:*Bacteria* | *Legionella* spp.:*Bacteria* |
| **1** |  | 0.468 ± 0.334 |  | 0.091 ± 0.157 | 0.001 ± 0.001 | 0.715 ± 0.011 |
| **2** |  | 0.676 ± 0.212 |  | n.d. | n.d. | 0.380 ± 0.021 |
| **3** |  | 24.208 ± 4.098 |  | 13.172 ± 1.511 | 0.053 ± 0.014 | 0.616 ± 0.063 |
| **4** |  | 27.705 ± 2.941 |  | 19.420 ± 2.286 | 0.107 ± 0.010 | 0.732 ± 0.023 |
| **5** |  | 0.760 ± 0.293 |  | 17.636 ± 6.261 | 0.059 ± 0.025 | 0.517 ± 0.064 |
| **6** |  | 1.025 ± 0.312 |  | 1.806 ± 0.999 | 0.007 ± 0.004 | 0.723 ± 0.042 |
| **7** |  | 4.785 ± 1.261 |  | 3.217 ± 3.339 | 0.004 ± 0.004 | 0.455 ± 0.425 |
